# Human stem cells alter the invasive properties of somatic cells via paracrine activation of mTORC1

**DOI:** 10.1038/s41467-017-00661-x

**Published:** 2017-09-19

**Authors:** Margit Rosner, Ha Thi Thanh Pham, Richard Moriggl, Markus Hengstschläger

**Affiliations:** 1 Institute of Medical Genetics, Center of Pathobiochemistry and Genetics, Medical University of Vienna, 1090 Vienna, Austria; 2 Ludwig Boltzmann Institute for Cancer Research, 1090 Vienna, Austria; 3 Institute of Animal Breeding and Genetics, University of Veterinary Medicine, 1210 Vienna, Austria; 4 Medical University of Vienna, 1090 Vienna, Austria

## Abstract

Controlled invasion is essential during many physiological processes, whereas its deregulation is a hallmark of cancer. Here we demonstrate that embryonic, induced pluripotent and amniotic fluid stem cells share the property to induce the invasion of primary somatic cells of various origins through insulin-like growth factor I (IGF-I)- or II (IGF-II)-mediated paracrine activation of mechanistic target of rapamycin complex 1 (mTORC1). We propose a model in which downstream of mTORC1 this stem cell-induced invasion is mediated by hypoxia-inducible factor 1-alpha (HIF-1α)-regulated matrix metalloproteinases. Manipulating the IGF signalling pathway in the context of teratoma formation experiments demonstrates that human stem cells use this mechanism to induce invasion and thereby attract cells from the microenvironment in vivo. In this study we have identified a so far unknown feature of human stem cells, which might play a role for the development of stem cell-derived tumours.

## Abstract (web summary)

Cell invasion is required for several physiological processes but it is unknown if stem cells induce invasiveness in other cells. Here, the authors show that human stem cells secrete insulin-like growth factor, which in turn activates the mTORC1 pathway, initiating invasive behaviour and attracting other cells.

## Introduction

For embryonic cells during development and for adult cells during tissue homoeostasis and repair, the ability to become motile and to navigate through extracellular matrix (ECM) is essential. This capacity to penetrate matrix barriers, defined as invasiveness, enables cells to spread within tissues, to cross basement membranes, and to enter and exit the vasculature to infiltrate neighbouring tissues and distant organs^1, 2^. The same strategies are co-opted by tumour cells during malignant transformation to invade adjacent tissues and metastasise to different organs. Furthermore, the directed movement of cells in response to extracellular cues drives their recruitment to specific sites where they fulfil their functions. The cell’s local microenvironment is a rich source of paracrine signals that direct cell fate including motility. Thus, cellular secretomes are pivotal mediators of cell–cell communication and fate conversion under both, physiologic and pathologic conditions^3, 4^.

Human pluripotent stem cells, such as embryonic stem cells (ESCs)^5^ and induced pluripotent stem cells (iPSCs)^6–8^, express a defined set of markers, proliferate indefinitely while maintaining cellular identity (self-renewal), are able to differentiate into cells of all three embryonic germ layers, and have the capacity to form teratomas upon injection into mice^9^. Due to their differentiation potential, these cells have widespread applications including disease modelling and cell replacement therapy^10, 11^. In contrast to the epithelial ESCs and iPSCs, mesenchymal human amniotic fluid stem cells (AFSCs) do not fulfil the entire spectrum of pluripotency criteria^12, 13^. To harness the full potential of stem cells upon identification of the putative limits and risks associated with their usage, it is mandatory to perform comparative studies including different types of stem cells. Without doubt, the thorough characterisation of stem cells at the molecular and functional level is an indispensable requirement for the decision on the ʽoptimal’ biological tool for use^14^.

Although paracrine communication between the cells of the inner cell mass and the trophectoderm or between extraembryonic and epiblast cells has been shown to be fundamental to early embryonic development^15, 16^, stem cell-derived paracrine signalling is still poorly investigated. Whereas a wide spectrum of factors have been tested with regard to their ability to affect stem cell potentials^17, 18^, secretome-associated functions of in vitro propagated human stem cells are barely studied.

To translate extracellular signals into coordinated intracellular actions, signal-integrating kinases are required. A vital sensor of multiple signalling inputs including growth factors is the kinase mTOR, which exists in two distinct multiprotein complexes, mTORC1 and mTORC2^19, 20^. In this study, we show that ESCs, iPSCs and AFSCs harbour the potential to induce invasion of various types of primary target cells in vitro via IGF-I- or IGF-II-mediated activation of mTORC1. We present results in favour of a model, in which HIF-1α-regulated matrix metalloproteinases (MMPs) transmit this function downstream of mTORC1. Application of human stem cells in the context of teratoma formation assays in mice confirmed that stem cell-induced somatic cell invasion also occurs in vivo. Adjacent to the teratoma, induction of invasiveness becomes detectable, accompanied by the activation of both, mTOR and MMPs. This is associated with the recruitment of murine cells into the teratoma. The spectrum of attracted targets includes cells expressing the haematopoietic lineage cell-specific protein 1 (HS1), the stromal marker smooth muscle actin (SMA) or the endothelial marker cluster of differentiation 31 (CD31). This recruitment from the microenvironment is diminished upon depletion of IGF-I or IGF-II in the stem cells. Under these experimental conditions, tumour size is reduced without effects on teratoma differentiation or apoptosis. In summary, this study reports the identification of a so far undescribed paracrine potential of human stem cells.

## Results

### Human stem cells induce invasion of somatic cells in vitro

**Fig. 1:**
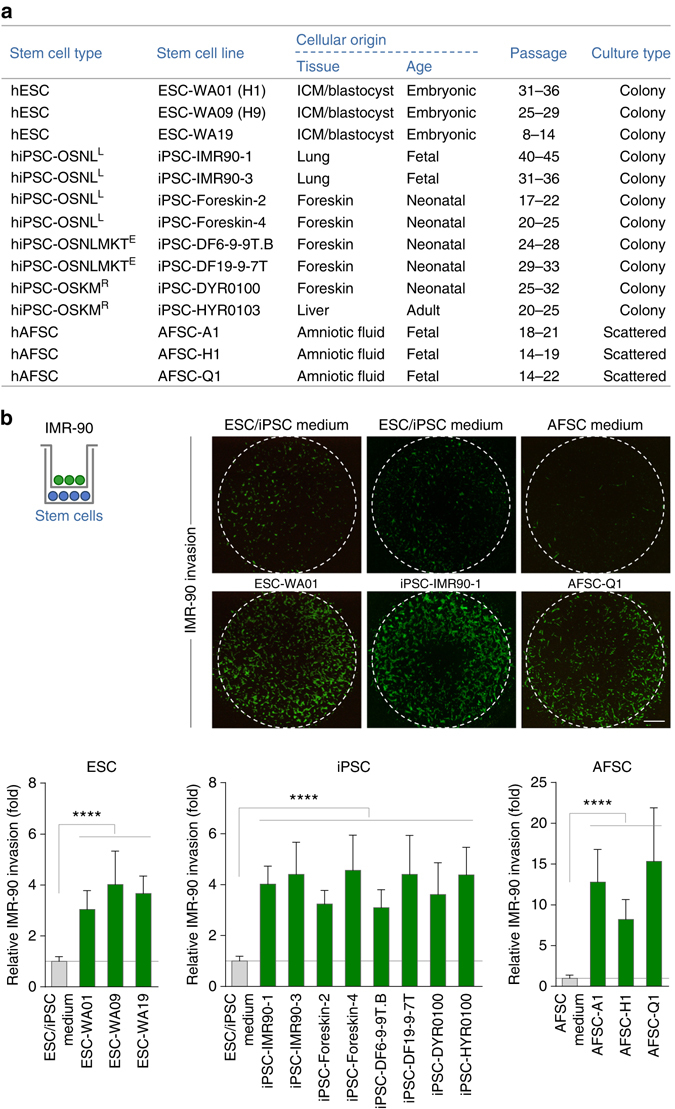
Human stem cells induce the invasion of primary fibroblasts. **a** Human embryonic (ESC), induced pluripotent (iPSC) and amniotic fluid (AFSC) stem cells analysed in this study. Passage numbers indicate the range within which experiments were performed. OSNL^L^, OCT4, SOX2, NANOG, LIN28, lentiviral transduction; OSNLMKT^E^, OCT4, SOX2, NANOG, LIN28, c-MYC, KLF4, SV40LT, episomal transduction; OSKM^R^, OCT4, SOX2, KLF4, c-MYC, retroviral transduction. **b** Transwell invasion assay of IMR-90 fibroblasts co-cultured with ESCs, iPSCs or AFSCs. Pictures show representative Calcein-stainings of invasive IMR-90 cells upon stem cell co-culture. Data are presented as the fold change of the corresponding medium control (*n* ≥ 6; mean ± s.d.). *Scale bar*, 800 μm. ****P < 0.0001 by unpaired, two-tailed Student’s *t*-test analysis. *n* refers to biological replicates

For the comparative study of stem cells presented here, we assembled a panel of human well-studied stem cell lines at early to late passages, including three pluripotent ESCs and three different mesenchymal AFSCs. In case of iPSCs we used eight different cell lines covering different reprogramming approaches^21^ (Fig. 1a and ‘Methods’ section). To investigate stem cell-mediated effects on the invasive behaviour of target cells we decided to use the well-established transwell invasion assay^22^ for the following reasons. In the bottom chamber, all different stem cell lines can be grown in their individual culture medium under maintenance conditions. In the course of this study, the undifferentiated status of the used stem cell lines during maintenance and experimentation, including the transwell assay, has routinely been proven via immunofluorescence and immunoblotting of stem cell markers (see e.g. Supplementary Fig. 5d). This highly reproducible and sensitive assay allows the accurate detection of as little as one invasive cell out of 2.5 × 10^4^ cells. Moreover, the separated chambers create optimal conditions to apply growth factor gradients for chemotactic studies. The use of 24- or even 96-well assay plates enabled us to compare the effects of a large number of secretomes of different cells or different treatments under identical experimental settings. In vitro transwell assays were evaluated after 22 h to optimise the chance to detect invasion induced by all studied stem cell types while minimising the risk for secondary effects due to long-term incubation.

Performing this assay, we found that co-culture of all studied stem cell lines dramatically induced the invasion of human IMR-90 fibroblasts (Fig. 1b). IMR-90 are primary fibroblasts isolated from non-diseased pulmonary tissue ideally fulfilling the requirements of candidate target cells. They harbour a normal diploid karyotype, are non-immortalised, are non-transformed, are genetically stable and are barely invasive (Fig. 1b)^23, 24^. Since IMR-90 cells have a doubling time of 48–72 h^23, 24^ our finding that stem cell-induced invasion was already detectable after 12 h (Supplementary Fig. 1a) proves this phenomenon to occur independently of proliferative effects. Moreover, we found that stem cell-conditioned medium (CM) was sufficient to trigger IMR-90 invasion, demonstrating that stem cells induce invasion via releasing stable factor(s) (Supplementary Fig. 1b).

**Fig. 2:**
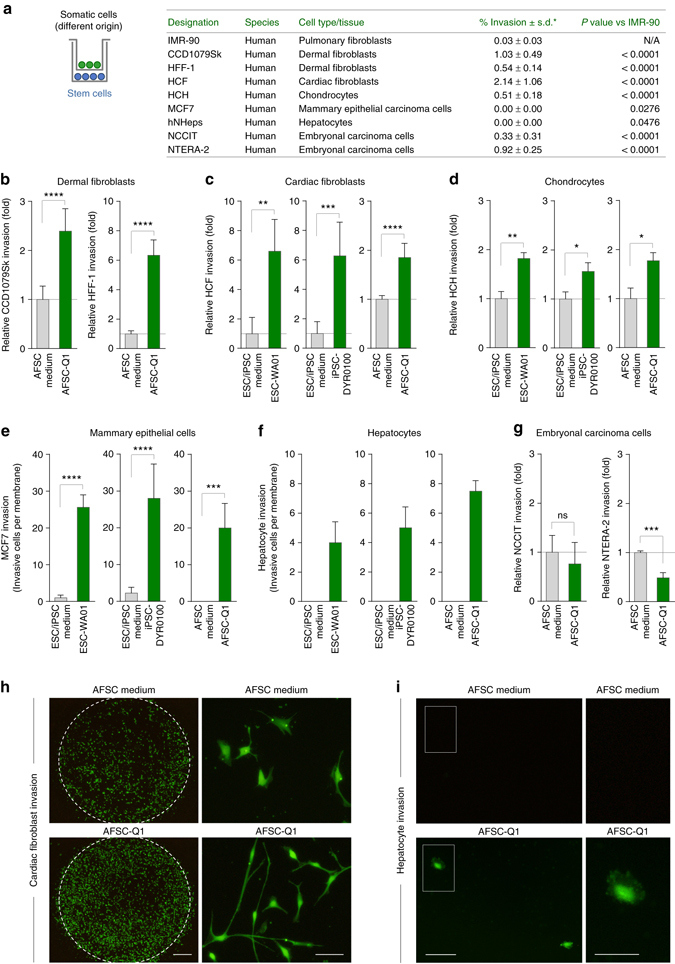
Human stem cells trigger the invasion of various somatic cell types. **a** Outline of the invasion experiments in **b**–**i**. The table summarises the analysed target cells and their mean invasive capacity. *Invasion data are given as the percentage of invasive cells evaluated via gradient-based transwell invasion assay (*n* ≥ 3; mean ± s.d.). N/A, not applicable. **b**–**g** Transwell invasion assay of indicated target cells upon co-culture with stem cells. Data are presented as the fold change of the corresponding medium control or the absolute number of invasive cells per membrane (*n* ≥ 3; mean ± s.d.). **h** Calcein-staining of invasive cardiac fibroblasts upon AFSC co-culture representative of data in **c** *. Scale bar*, *left panels*, 800 μm; *right panels*, 100 μm. **i** Calcein-staining of invasive hepatocytes upon AFSC co-culture representative of data in **f**. *Scale bar*, *left panels*, 100 μm; *right panels*, 50 μm. *P < 0.05; **P < 0.01; ***P < 0.001; ****P < 0.0001; ns, *P* > 0.05 (not significant) by unpaired, two-tailed Student’s *t*-test analysis. *n* refers to biological replicates

To characterise this stem cell property in more detail, we performed co-culture experiments with somatic cells highly diverse in terms of their tissue origin and invasive capacity (Fig. 2a). Similar to IMR-90, primary mesenchymal cells of different origin and varying invasive abilities including dermal fibroblasts, adult cardiac fibroblasts and femoral chondrocytes all showed enhanced invasiveness upon co-culture with stem cells (Fig. 2a–d, h). Next, to see whether ESCs, iPSCs and AFSCs might also have the potential to confer invasiveness to entirely non-invasive cells, we tested the well-established model of MCF7 mammary cells (Fig. 2a, e)^25^. In addition to mammary epithelial cells, we found that non-invasive primary hepatocytes could also be activated to cross the matrix when co-cultured with stem cells (Fig. 2a, f, i). On the other hand, our observation that embryonic carcinoma cell invasion could not be further induced (Fig. 2a, g), demonstrated that stem cell co-culture triggers a pro-invasive response depending on the individual cellular background and does not cause invasion-supporting artefacts (Fig. 2a, g). Overall, these results prove that ESCs, iPSCs and AFSCs share the property to activate somatic cell invasion in a paracrine manner.

### Human pluripotent stem cells induce invasion in vivo

**Fig. 3:**
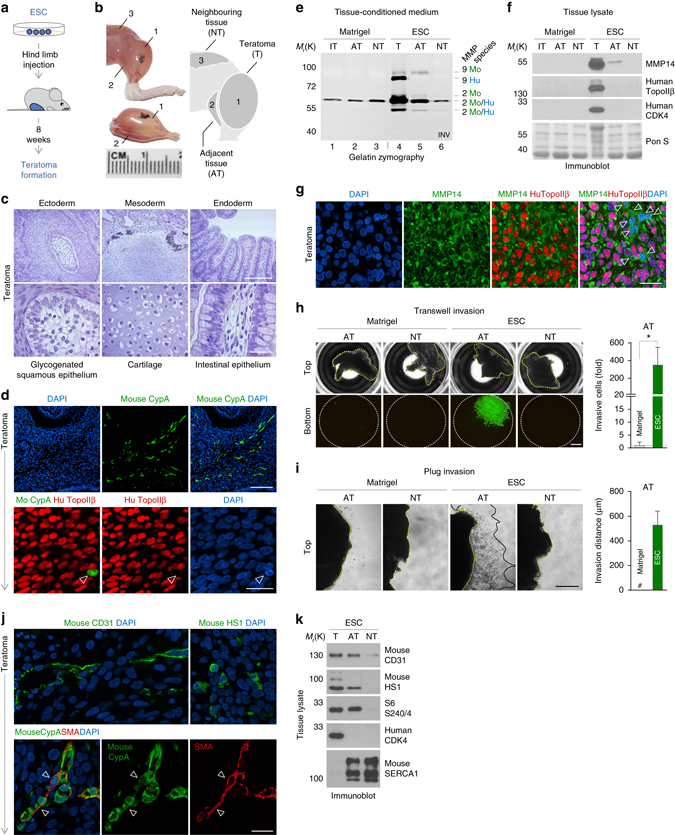
Stem cells trigger invasion in vivo. **a** Outline of the experiments in **b**–**k**. **b** Illustration of tissue sampling. Numbers indicate collected tissues. **c** H&E staining of teratomas to detect three germ layer-differentiation. *Scale bars*, 100 and 20 μm. **d** Immunostaining of teratoma tissue for the detection of mouse cyclophilin A (*upper panels*) or mouse cyclophilin A and human topoisomerase IIβ (*lower panels*). Nuclei were counterstained with DAPI. The *arrrowhead* indicates a mouse cell within human teratoma cells. *Scale bars*, 150 and 20 μm. **e** Gelatin zymography of tissue-conditioned medium from Matrigel- or ESC-injected mice to detect secreted MMPs. The gel picture was colour-inverted. The bar indicates vertical cropping. IT, injected tissue. **f** Immunoblot of tissue lysates for the detection of MMP14. The absence of human cells from non-teratoma tissues and equal loading was verified by reprobings with human-specific antibodies and Ponceau-S staining. **g** Immunostaining of teratoma tissue for the detection of MMP14 and human topoisomerase IIβ. Nuclei were counterstained with DAPI. *Arrowheads* indicate mouse cells within human teratoma cells. *Scale bar*, 20 μm. **h** Ex vivo transwell invasion assay of indicated tissues. Pictures show the tissues in the top chambers, and the invasive, Calcein-stained cells on the bottom membranes. Data are presented as the fold change of the Matrigel control (*n* = 4; mean ± s.d.). *Scale bar*, 1 mm. **i** Ex vivo plug invasion assay of indicated tissues. Pictures show the tissues on the top of the plug (*yellow line*) and their invasive outgrowth (*grey line*). Data represent the invasion distance in μm (*n* = 4; mean ± s.d.). #, non-detectable. *Scale bar*, 400 μm. **j** Immunostaining of murine CD31 (*upper left panel*), HS1 (*upper right panel*) and SMA (*lower panels*) in teratoma tissue. Mouse-specific SMA expression was detected via Cyclophilin A-co-staining. *Arrowheads* denote a fibroblast-like, SMA + murine cell. *Scale bar*, 20 μm. **k** Immunoblot of tissue lysates from ESC-injected mice for the detection of indicated proteins. The absence of mouse tissue from teratoma lysates was verified by analysing murine SERCA1. *P < 0.05 by unpaired, two-tailed Student’s *t*-test analysis. *n* refers to biological replicates

To test whether stem cell-induced invasion also occurs in vivo, we performed teratoma formation assays upon implantation of human ESCs into mice. In all presented in vivo experiments, matrigel injections served as a negative control for the stem cell-mediated effects in the host. In 8 weeks’ fully differentiated teratomas as well as in not-fully differentiated teratoma tissues 4 weeks after stem cell implantation, the appearance of murine cells identified by a mouse-specific cyclophilin A antibody was detected (Fig. 3a–d and Supplementary Fig. 2a–c). Gelatin zymography, immunoblot and immunofluorescent analyses demonstrated the existence of murine cells with induced expression of MMP2, MMP9 or MMP14 in the teratoma (T) and in muscle (gastrocnemius) tissue adjacent to the teratoma (adjacent tissue, AT), but not in distant muscle (vastus) tissue with low teratoma proximity (neighbouring tissue, NT) (Fig. 3b, e–g and Supplementary Fig. 2d). Ex vivo transwell and plug invasion assays using collected tissues revealed that this MMP induction was accompanied by the initiation of invasion in adjacent, but not in neighbouring tissue (Fig. 3h, i). Using immunostaining and immunoblotting at the same time points of teratoma development the spectrum of attracted targets was shown to include cells expressing the haematopoietic marker HS1, the stromal marker SMA or the endothelial marker CD31 (Fig. 3j, k and Supplementary Fig. 2e, f). It is already well established, that in the context of tumour development HS1-positive cells are recruited from the peripheral blood via activation of transendothelial migratory potential. Moreover, SMA expression is induced in adjacent fibroblasts triggering their invasive status and CD31-positive cells are recruited via activation of their invasive behaviour. Recruitment of these cell types is known to support tumour growth via secreting relevant growth factors and inducing angiogenesis^26–28^.

In conclusion, these results show that first, stem cells induce invasion of non-invasive murine tissue adjacent to the teratoma and that second, murine cells invade into the teratoma, proving stem cell-mediated invasion to also occur in vivo. Third, this initiation of invasion is accompanied by the induction of matrix-degrading proteases.

### Stem cell-induced invasion is mTOR-dependent

**Fig. 4:**
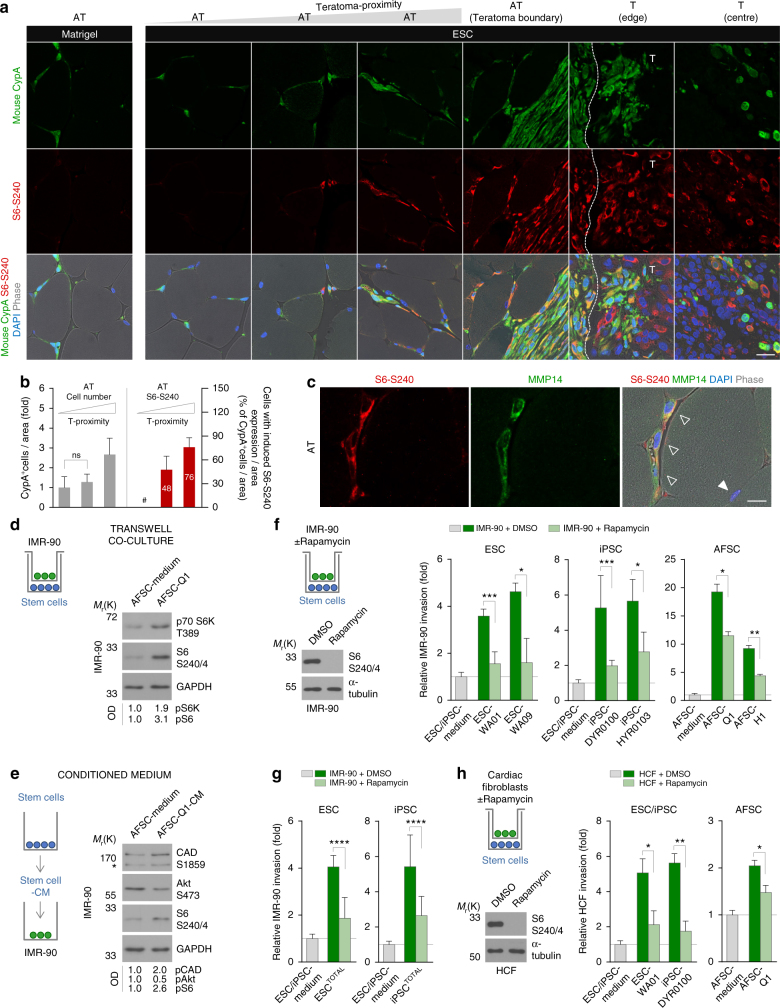
Stem cells trigger target cell invasion via paracrine activation of mTORC1. **a** Immunostaining of indicated tissues from Matrigel- or ESC-injected mice for the detection of mouse cyclophilin A and phosphorylated S6. Nuclei were counterstained with DAPI. The *dotted line* in the ʽT (edge)’ panel delineates the border between adjacent and teratoma (T) tissue. *Scale bar*, 20 μm. **b** Quantification of cyclophilin A+ cells with induced S6 phosphorylation in the adjacent tissue of ESC-injected mice (*n* ≥ 7; mean ± s.d.). Data are based on immunostainings as presented in **a**. #, non-detectable. **c** Immunostaining of adjacent tissue from ESC-injected mice for the detection of phosphorylated S6 and MMP14. Nuclei were counterstained with DAPI. Pictures show double-positive (*open arrowheads*) and double-negative (*filled arrowhead*) murine cells. *Scale bar*, 10 μm. **d** Immunoblot for the detection of mTOR target proteins in IMR-90 cells co-cultured with AFSCs for 2 h. Data were densitometrically evaluated (OD). **e** Immunoblot for the detection of mTOR target proteins in IMR-90 cells treated with AFSC-conditioned medium for 1 h. Data were densitometrically evaluated (OD). The asterisk next to the CAD S1859 panel indicates a non-specific band. **f** Transwell invasion assay of IMR-90 fibroblasts treated with Rapamycin and co-cultured with stem cells (*n* ≥ 4; mean ± s.d.). The efficiency of Rapamycin treatment under the chosen assay conditions was evaluated via immunoblotting of phosphorylated S6. **g** The experiment in **f** was performed for all ESC and iPSC lines described in Fig. 1a. Results are presented in groups summarising the data for all ESC and iPSC lines including data in **f** (*n* = 12, ESC^TOTAL^; *n* = 38, iPSC^TOTAL^; mean ± s.d.). **h** Transwell invasion assay of Rapamycin-treated cardiac fibroblasts upon stem cell co-culture and immunoblot of phosphorylated S6, as performed for IMR-90 in **f** (*n* ≥ 3; mean ± s.d.). *P < 0.05; **P < 0.01; ***P < 0.001; ****P < 0.0001; ns, *P* > 0.05 (not significant) by unpaired, two-tailed Student’s *t*-test analysis. *n* refers to biological replicates

Secretomes are rich sets of molecules that once released from the cell, impinge on the microenvironment to induce specific biochemical cascades which regulate biological properties such as target cell invasion. To test whether one of the most potent intracellular mediators of paracrine signals, the IGF/mTOR cascade, could be involved in the here described stem cell-induced invasion we analysed the expression of phosphorylated S6 protein, which is the best described and most used readout for activation of mTORC1^19, 20^. In the course of teratoma formation 4 weeks and 8 weeks after ESC injection, we observed the induction of mTORC1 activity in murine cells in the adjacent tissue and in the teratoma, but not in the neigbouring tissue (Fig. 3k and Fig. 4a and Supplementary Fig. 3a, b). We found an inverse correlation between the amount of cells with activated mTORC1 and the distance to the tumour, what supports the notion that activation of mTORC1 could be mediated by the pluripotent stem cells (Fig. 4a). In the area with lower proximity to the teratoma, we found a remarkable induction of mTORC1-activated cells without a significant increase of total cell numbers suggesting the activation of tissue resident cells. Close to the teratoma, 76% of all murine cells were detected to be positive for mTORC1 activity, what was accompanied by an about 2.5-fold induction of the total cell number indicating the recruitment of additional cells invading from other sources (Fig. 4a, b). The fact that activation of mTORC1 in cells of the adjacent tissue correlated with the intracellular induction of MMP14 expression (Fig. 4c) suggested that the activation of this kinase and the induction of invasion might be associated and prompted us to further investigate this putative association.

If the mTOR kinase pathway is indeed involved in the here described phenomenon one would expect to see an intracellular induction of this cascade under conditions of stem cell-induced invasion in vitro. In agreement with this notion, immunoblotting demonstrated that stem cell co-culture and stem cell-CM caused the paracrine activation of mTORC1 target proteins in IMR-90 cells (Fig. 4d, e). Rapamycin treatment of IMR-90 fibroblasts during co-culture with ESCs, iPSCs and AFSCs revealed that the specific inhibition of mTORC1 caused a significant reduction in stem cell-induced invasion (Fig. 4f and Supplementary Fig. 3c–e). Importantly, these results could be confirmed for all ESC and iPSC lines and recapitulated using cardiac fibroblasts (Fig. 4g, h and Supplementary Fig. 3c, f, g).

**Fig. 5:**
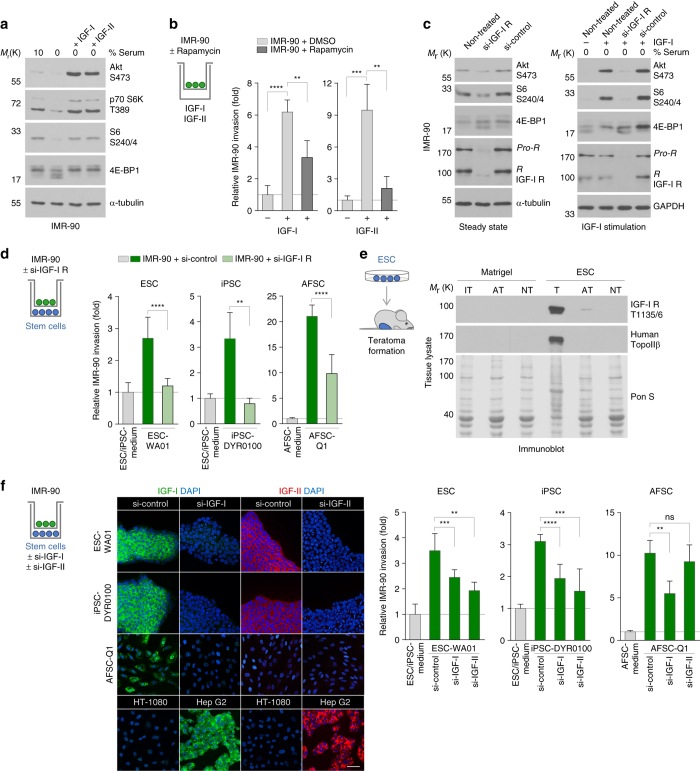
Stem cell-secreted IGFs activate the IGF-I receptor to promote target cell invasion. **a** Immunoblot for the detection of mTOR signalling proteins in serum-deprived IMR-90 cells stimulated with IGF-I or IGF-II. **b** Transwell invasion assay of Rapamycin-treated IMR-90 fibroblasts upon IGF stimulation (*n* ≥ 3; mean ± s.d.). **c** Immunoblots for the detection of mTOR signalling proteins in IGF-I receptor-depleted IMR-90 cells under steady state conditions (*left panel*) or upon IGF-I stimulation (*right panel*). **d** Transwell invasion assay of IGF-I receptor-depleted IMR-90 fibroblasts upon co-culture with stem cells (*n* ≥ 6; mean ± s.d.). **e** Immunoblot of tissue lysates from Matrigel- or ESC-injected mice for the detection of phosphorylated IGF-I receptor. The absence of human cells from non-teratoma tissues and equal loading was verified by detection of human topoisomerase IIβ and Ponceau-S staining. **f** Transwell invasion assay of IMR-90 fibroblasts co-cultured with IGF-I- or IGF-II-depleted stem cells (*n* ≥ 3; mean ± s.d.). Knockdown efficiency was evaluated via immunostaining of endogenous IGF-I and IGF-II. IGF detection in stem cells was further verified by co-analysing somatic (HT-1080, Hep G2) control cell lines. *Scale bar*, 50 μm. **P < 0.01; ***P < 0.001; ****P < 0.0001; ns, *P* > 0.05 (not significant) by unpaired, two-tailed Student’s *t*-test analysis. *n* refers to biological replicates

IGFs are potent activators of mTOR known to be expressed in and released from ESCs and AFSCs^17, 29, 30^. To test their role in stem cell-induced invasion, we first demonstrated that human recombinant IGF-I and IGF-II were sufficient to trigger mTORC1 activity and to induce invasion of target cells in a Rapamycin-sensitive manner, proving the role of mTORC1 (Fig. 5a, b and Supplementary Fig. 4a). We reasoned that if IGFs were indeed involved, inhibition of their receptor should impact target cell invasion. To exclude the compensatory action of other mTOR-inducing receptors, e.g. the insulin receptor, we verified that depletion of the IGF-I receptor actually blocked mTORC1 activity in the used target cell. Next to the reduced activation of mTORC1, inhibition of the IGF-I receptor caused a significant decrease in stem cell-induced IMR-90 invasion (Fig. 5c, d). The role of this receptor is further supported by our finding that in the adjacent tissue collected from stem cell-injected mice the activated, T1135/6 phosphorylated form of the IGF-I receptor could be detected (Fig. 5e).

To verify that stem cells indeed release IGFs as the receptor-activating ligands to induce target cell invasion, we performed IGF knockdown in ESCs, iPSCs and AFSCs. Co-culture experiments demonstrated that depletion of IGF-I in ESCs, iPSCs and AFSCs diminished their capacity to induce IMR-90 invasion. Since AFSCs do not express IGF-II, depletion of this factor only affected the stem cell-inducing potential of ESCs and iPSCs (Fig. 5f). The role of IGFs is further substantiated by the finding that blocking IGF-I using a neutralising antibody^31^ reduced the levels of stem cell-induced IMR-90 and MCF7 invasion (Supplementary Fig. 4b), and by the observation that cells negative for the expression of IGFs cannot induce invasion (Supplementary Fig. 4c). Finally, we found that—in the absence of an exogenous stimulus—induction of mTORC1 activity via siRNA-mediated depletion of its endogenous inhibitor TSC2 caused an increase in IMR-90 invasion in a MMP-dependent manner, corroborating the positive link between mTORC1, MMPs and invasiveness in these cells (Supplementary Fig. 4d–g).

Taken together, we present functional data demonstrating that human stem cells induce somatic cell invasion via paracrine activation of IGF/mTOR signalling.

### Stem cell-induced invasion in teratoma development

**Fig. 6:**
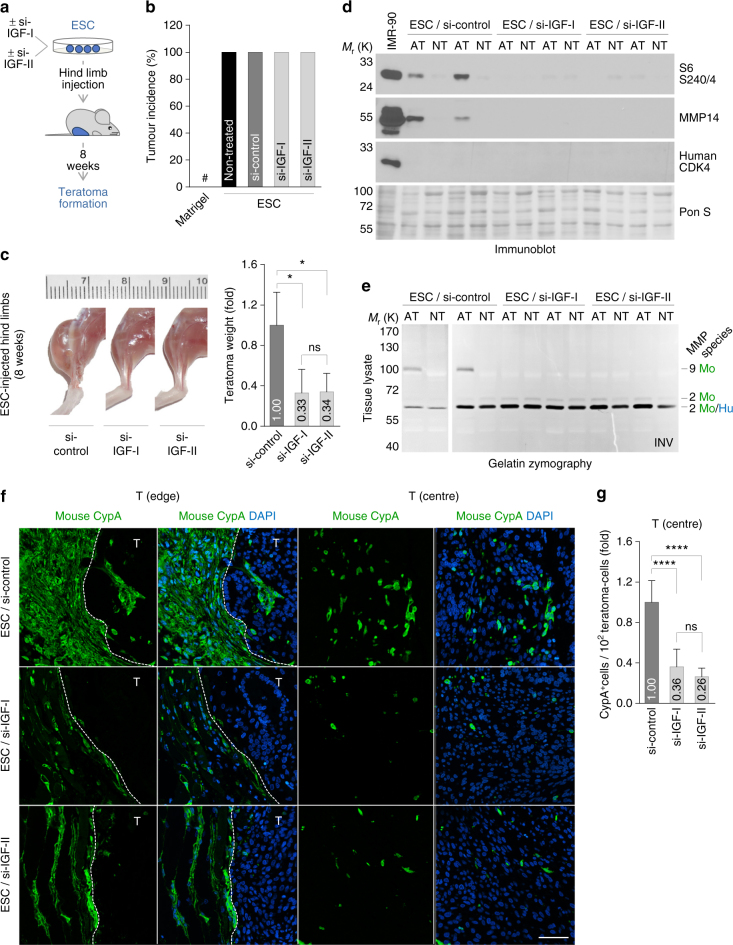
ESC-mediated target cell invasion contributes to teratoma growth. **a** Outline of the experiments in **b**–**g**. **b** Incidence of teratoma formation upon injection of IGF-I- or IGF-II-depleted ESCs. Matrigel-injected mice were co-analysed as controls. #, non-detectable (*n* = 4; mean ± s.d.). **c** Teratoma weight upon injection of IGF-I- or IGF-II-depleted ESCs. Representative pictures show ESC-injected hind limbs 8 weeks after implantation. Data are presented as the fold change of the non-targeting control (si-control) (*n* ≥ 3; mean ± s.d.). **d** Immunoblot of tissue lysates from ESC-si-control- or ESC-si-IGF-injected mice for the detection of phosphorylated S6 and MMP14. The absence of human cells from non-teratoma tissues and equal loading was verified by detection of human CDK4 and Ponceau-S staining. IMR-90 lysate was co-analysed as a positive control for the detection with human-specific antibodies. **e** Gelatin zymography of tissue lysates from ESC-si-control- or ESC-si-IGF-injected mice for the detection of MMPs. The gel pictures were colour-inverted. **f** Immunostaining of teratoma tissues from ESC-si-control- or ESC-si-IGF-injected mice for the detection of mouse cyclophilin A. Nuclei were counterstained with DAPI. The dotted line in the ʽT (edge)’ panel delineates the border between adjacent and teratoma (T) tissue. *Scale bar*, 50 μm. **g** Quantification of cyclophilin A-positive cells in teratomas from ESC-si-control- or ESC-si-IGF-injected mice. Data are based on immunostainings as shown in **f** and are presented as the fold change of the non-targeting control (si-control) (*n* ≥ 6; mean ± s.d.). *P < 0.05; ****P < 0.0001; ns, *P* > 0.05 (not significant) by unpaired, two-tailed Student’s *t*-test analysis. *n* refers to biological replicates

We next designed an experimental approach to investigate the role of stem cell-released IGFs for the induced invasion in vivo. First, we confirmed that human IGFs trigger comparable intracellular effects on the mTOR pathway in murine and human cells (Supplementary Fig. 5a, b). Human ESCs depleted of IGF-I or IGF-II were injected into mice and teratoma formation was monitored 8 weeks after implantation. Whereas the incidence of tumour formation was not affected, knockdown of IGF-I and IGF-II caused a significant reduction of teratoma size (Fig. 6a–c). Before implantation, depletion of IGFs was proven to neither affect apoptosis nor to alter the differentiation status of the ESCs used for teratoma formation (Supplementary Fig. 5c, d). As proven by H&E staining and immunoblotting, teratomas derived from IGF-depleted stem cells did not exhibit any alterations with regard to germ layer differentiation, stem cell marker expression or apoptosis (Supplementary Fig. 5e–g). Strikingly, we found significantly reduced induction of mTORC1, MMP14 and MMP9 in the adjacent tissue of teratomas derived from IGF-depleted stem cells (Fig. 6d, e). Furthermore, this was accompanied by a severe reduction of murine cells attracted into the teratoma (Fig. 6f, g).

Taken together, we report that depletion of endogenous IGFs in human pluripotent stem cells causes negative effects on teratoma growth accompanied by reduced activation of mTORC1 and MMPs in the tumour microenvironment and less attracted murine cells in the teratoma. This occurs without any affects on teratoma formation incidence, the differentiation status of the teratoma, or apoptosis. In conclusion, these findings provide evidence for a model, in which human stem cells recruit cells from the microenvironment, which can mediate supportive effects on tumour growth.

### HIF-1α and MMPs in mTOR-dependent stem cell-induced invasion

**Fig. 7:**
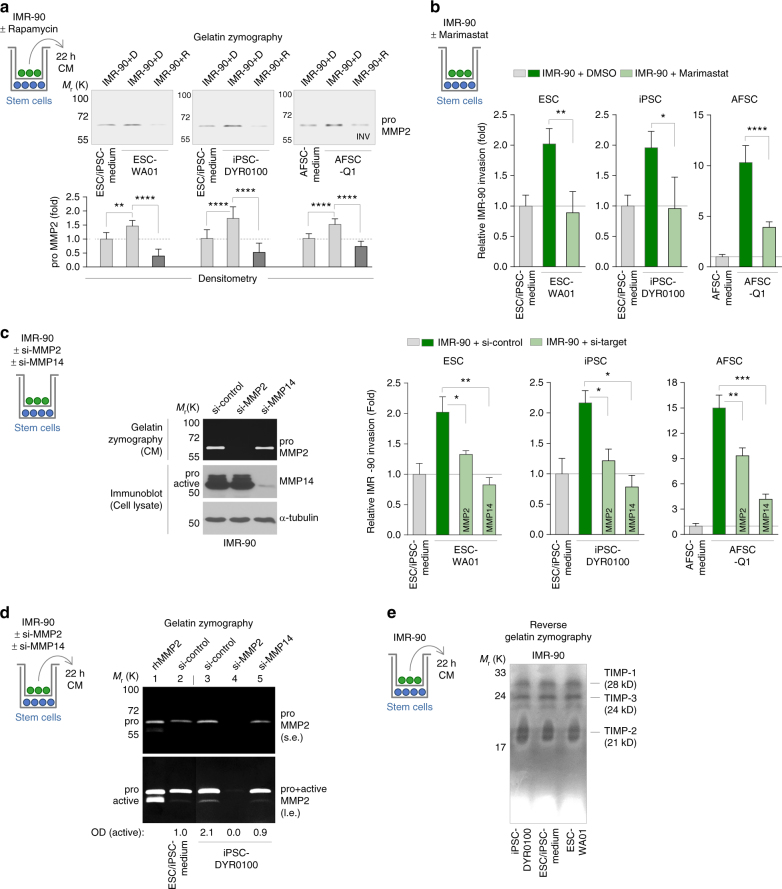
Stem cell-induced invasion is mediated by the mTORC1-dependent activation of MMPs. **a** Gelatin zymography of conditioned medium for the analysis of secreted MMP2 in Rapamycin-treated IMR-90 cells co-cultured with stem cells. Results of independent experiments were densitometrically analysed (*n* ≥ 6; mean ± s.d.). The gel pictures were colour-inverted. **b** Transwell invasion assay of IMR-90 fibroblasts treated with Marimastat and co-cultured with stem cells (*n* ≥ 3; mean ± s.d.). **c** Transwell invasion assay of MMP2- or MMP14-depleted IMR-90 fibroblasts upon co-culture with stem cells (*n* ≥ 3; mean ± s.d.). Knockdown efficiency was assessed via gelatin zymography and immunoblotting. **d** Gelatin zymography of conditioned medium for the analysis of secreted MMP2 in MMP2- or MMP14-depleted IMR-90 fibroblasts upon co-culture with iPSCs. Data were densitometrically evaluated (OD). The *bar* indicates vertical cropping. **e** Reverse gelatin zymography of conditioned medium for the analysis of secreted TIMPs in IMR-90 fibroblasts co-cultured with stem cells. *P < 0.05; **P < 0.01; ***P < 0.001; ****P < 0.0001 by unpaired, two-tailed Student’s *t*-test analysis. *n* refers to biological replicates

As described above, we found stem cell-induced invasion to correlate with the induction of MMPs, which are well known molecular executors of invasion via their matrix-degrading potential^32, 33^. We next wanted to investigate whether MMPs are indeed causatively involved in this phenomenon and whether their induction is under the control of mTORC1. We first performed zymography on the transwell top chambers-derived medium of the already presented experiments (Fig. 4f–h and Supplementary Fig. 4b) to show that stem cell-induced invasion of IMR-90 cells, cardiac fibroblasts and MCF7 cells was accompanied by the increased expression of secreted pro-MMP2 (Fig. 7a and Supplementary Fig. 6a, b). Our finding that stem cell-induced induction of MMP2 could be blocked by Rapamycin proves a functional link between mTORC1 and MMP2 induction (Fig. 7a). The fact that in these cells MMPs are under the control of mTORC1 is further supported by our finding that Rapamycin negatively affects MMP14 levels (Supplementary Fig. 6c). The proof that MMPs are essential for stem cell-induced invasion was obtained by the observation that neither ESCs, nor iPSCs or AFSCs could induce invasion of IMR-90 cells pre-treated with the broad spectrum MMP inhibitor Marimastat (Fig. 7b). The MMPs most frequently involved in the regulation of invasion are MMP14, MMP2 and MMP9^33^. siRNA-mediated depletion of endogenous MMPs revealed that in IMR-90 cells, which do not express MMP9 (Fig. 7a, c, d), the activation of MMP2 is under the control of MMP14 and their stem cell-induced invasion depends on both, MMP14 and MMP2 (Fig. 7c, d). The activity of MMPs is known to also be under the control of endogenous tissue inhibitors of metalloproteinases (TIMPs)^32, 33^. However, we found that human stem cells do not affect the expression of TIMP1, 2 and 3 in IMR-90 target cells (Fig. 7e). Taken together, these findings show that beside mTORC1, MMPs are essential regulators of the here reported stem cell-induced invasion.

**Fig. 8:**
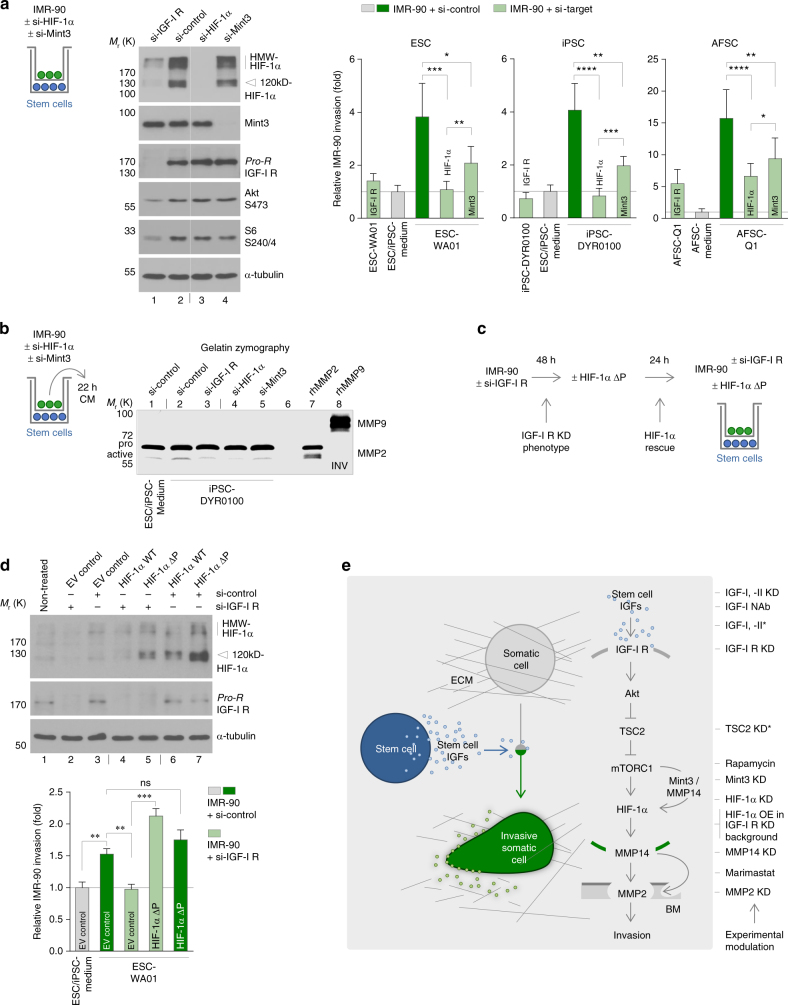
Stem cell-induced target cell invasion is HIF-1α dependent. **a** Transwell invasion assay of HIF-1α- or Mint3-depleted IMR-90 fibroblasts upon co-culture with stem cells. IGF-I receptor-depleted cells were co-analysed (*n* ≥ 5; mean ± s.d.). Knockdown efficiency and downstream effects were evaluated via immunoblotting. The bar indicates vertical cropping. **b** Gelatin zymography of conditioned medium for the analysis of secreted MMP2 in HIF-1α-, Mint3- or IGF-I receptor-depleted IMR-90 fibroblasts upon co-culture with iPSCs. The gel picture was colour inverted. The *bars* indicate vertical cropping. **c** Outline of the transfection experiment presented in **d**. **d** Transwell invasion assay of IGF-I receptor-depleted, HIF-1α ΔP overexpressing IMR-90 fibroblasts co-cultured with ESCs. Knockdown cells expressing ectopic HIF-1α ΔP were identified via co-transfection of GFP-Spectrin (*n* = 3; mean ± s.d.). HIF-1α overexpression in IGF-I receptor-depleted cells was verified via immunoblotting. The *bar* indicates vertical cropping. **e** Proposed mechanism of stem cell-induced invasion. The scheme includes a summary of the underlying experimental approaches. *Asterisks* indicate approaches to prove IGF/mTORC1-dependence of target cell invasion in the absence of stem cell co-culture. KD, knockdown; NAb, neutralising antibody; OE, overexpression; BM, basement membrane. *P < 0.05; **P < 0.01; ***P < 0.001; ****P < 0.0001; ns, *P* > 0.05 (not significant) by unpaired, two-tailed Student’s *t*-test analysis. *n* refers to biological replicates

All presented data on the pivotal role of mTORC1 and MMPs described above raise the question how mTORC1 can regulate the expression of MMPs in the context of stem cell-induced invasion. To our best knowledge, so far a gapless biochemical intracellular cascade for the signal transmission from the paracrine activation of the IGF-I receptor to the regulation of MMPs has not been suggested. Even less is known about the putative role of such a cascade for cell invasion. Still, although in this study the involved mediators remained elusive, at least a link between IGF-I receptor activity and MMP2 mRNA and protein expression was described in murine lung cancer cells^34^. Being aware of the fact that downstream of mTORC1 many different molecules have been reported, which could be involved in the here described process, we still deemed it important to provide a more detailed picture of mTOR-mediated MMP regulation. Next to its function as global translational regulator, mTORC1 is known to control the activity of transcription factors^19, 20^. Among these, the transcription factor HIF-1α triggered our attention, because it is known for its role in the regulation of MMP expression. Under normoxia, HIF-1α is rapidly degraded, what is counteracted by mTORC1-mediated upregulation of its translation. Furthermore, the transcriptional activity of HIF-1α is enhanced by Mint3. This function of Mint3 has been demonstrated to depend on its mTORC1-triggered phosphorylation and on its co-factor MMP14. Accordingly, mTORC1 activates HIF-1α via two mechanisms^35, 36^. As a next step, we investigated the regulation and interdependence of these players in the somatic target cells. Using different experimental settings, we found that recombinant IGF-I upregulates HIF-1α, what can be blocked by Rapamycin (Supplementary Fig. 7a), knockdown of the IGF-I receptor downregulates HIF-1α (Supplementary Fig. 7b), knockdown of HIF-1α or Mint3 downregulates activated MMP14 (Supplementary Fig. 7c), overexpression of HIF-1α upregulates activated MMP14 (Supplementary Fig. 7d, e), knockdown of Mint3 downregulates the expression of HIF-1α target genes (Supplementary Fig. 7f), knockdown of the IGF-I receptor, HIF-1α or the mTORC1 component Raptor downregulates activation of MMP2 (Supplementary Fig. 7g–k), and knockdown of the mTORC1 inhibitor TSC2 upregulates activated MMP2 in target cells (Supplementary Fig. 7g–k). Putting all these results together allows to draw a model, in which MMP expression in IMR-90 is under the control of mTORC1-regulated HIF-1α. Next, we wanted to obtain further insights into the role of this axis for stem cell-induced invasion. We show that the ESCs-, iPSCs- and AFSCs-induced invasion was significantly impaired in somatic target cells depleted of HIF-1α or Mint3 (Fig. 8a). Zymography on the transwell top chambers-derived conditioned medium of right these experiments revealed that depletion of HIF-1α or Mint3 not only impaired stem cell-induced invasion but also significantly diminished the stem cell-induced induction of MMP2 in these cells (Fig. 8a, b). Within the same experimental setting we also confirmed the negative effects of IGF-I receptor knockdown on stem cell-induced invasion (Fig. 8a) and demonstrated that stem cells cannot induce MMP2 expression upon IGF-I receptor depletion (Fig. 8b). Finally, we demonstrate that overexpression of stable HIF-1α can restore invasion of target cells, in which stem cell-induced invasion is impaired via IGF-I receptor depletion. Interestingly, overexpression of stable HIF-1α cannot hyperactivate stem cell-induced invasion, implicating that stem cells already fully activate the pro-inavsive potential of HIF-1α in these cells (Fig. 8d). In summary, these results demonstrate that HIF-1α and MMPs are essential mediators of stem cell-induced invasion, and allow to propose a model for the functional interdependence of the IGF-I receptor/mTORC1/HIF-1α/MMP axis in translating extracellular, stem cell-derived IGFs into invasive properties in the target cells (Fig. 8e).

## Discussion

In this study, we report a set of functional experiments demonstrating that human ESCs, iPSCs and AFSCs induce the invasion of somatic cells in vitro via IGF-mediated, paracrine activation of mTORC1. In total, six out of six studied human target cells harbouring low or undetectable invasive potential including primary cells of various origins have been proven to be susceptible to stem cell-induced invasion. Moreover, we demonstrate that stem cells cannot induce invasion of target cells depleted of HIF-1α or MMPs, pointing out that these molecules are essential for this paracrine stem cell function. Finally, we present various different experimental results supporting a model, in which HIF-1α-regulated MMPs mediate invasion downstream of mTORC1. Although the functional interdependence of this axis could not quintessentially be depicted and other mediators downstream of mTORC1 might also be involved, we feel this proposed model to be strongly supported by our data (Fig. 8e).

Furthermore, we performed in vivo experiments demonstrating that the application of human pluripotent stem cells and teratoma formation trigger induction of the mTOR pathway and activation of MMPs in mouse cells in the muscle tissue adjacent to the teratoma. Using ex vivo invasion assays we could demonstrate that this adjacent tissue becomes invasive. Murine cells were recruited into the teratoma and were shown to exhibit both, activated mTOR signalling and induced expression of MMPs. Depletion of IGF-I or IGF-II in the stem cells used for teratoma assays caused a decrease of mTOR and MMP activation in the adjacent tissue and triggered a smaller proportion of murine cells in the teratomas. Taken together, these findings confirm that the in vitro discovered stem cell-induced somatic cell invasion also occurs in vivo.

So far, studies evaluating the impact of stem cells on other cells are limited and focus on the interaction with cancer-derived cells^37, 38^. However, in general, intercellular paracrine communication, involving e.g. cells of the inner cell mass, has been shown to be fundamental to early embryonic development^15, 16^, and in particular, a role for paracrine IGF-I receptor signalling during murine preimplantation development has been proposed^39^. Accordingly, the findings on stem cell-induced invasion presented here could open a new field of investigation on the in vivo relevance of paracrine stem cell functions in a non-tumourigenic context, such as, e.g. during human embryogenesis.

It is well known that tumour growth and progression do not solely depend on cancer cell-autonomous defects, but are also under the control of the tumour microenvironment. Tumour-derived paracrine signals, such IGF-I or IGF-II, activate cell migration and invasion and have been implicated in the recruitment of different cells from the microenvironment^27, 40^. For example, this paracrine process activates and recruits cancer associated fibroblasts, which express SMA and migrate in an MMP-dependent manner. So activated fibroblasts are believed to regulate cancer progression via their active secretome including growth factors^28^. IGF-I and IGF-II have been demonstrated to drive the recruitment of endothelial cells in the context of angiogenesis, which is essential for tumour growth and metastasis^41^. Cancer cell-secreted IGF-II has recently been shown to induce SMA expression and invasion in fibroblasts and to drive the functional incorporation of fibroblasts and bone-marrow-derived vascular progenitor cells into primary tumours to facilitate tumour growth^42^. We here demonstrate for the first time that human stem cells induce invasion of somatic cells via IGF-I- or IGF-II-mediated activation of mTOR. Furthermore, we found, that in the course of in vivo teratoma formation assays human stem cells make use of this mechanism to recruit murine cells. Mouse cells invaded into the tumour express the haematological marker HS1, the stromal marker SMA or the endothelial marker CD31. As described above, these invaded cell types are known to support tumour growth, via affecting different tumour-relevant processes including e.g. angiogenesis. Teratoma development is defined as the differentiation of stem cells into various cell types of all three germ layers followed by proliferation of differentiated cells. Since it has been demonstrated, that in vivo injected undifferentiated stem cells do not proliferate, teratoma growth reflects the proliferative activity of differentiated cells^43^. In this study, we found that inhibition of the described paracrine stem cell function via depletion of endogenous IGFs (1) did not have any affects on teratoma formation incidence, (2) significantly reduced tumour size, (3) did not have any affects on the differentiation status of the teratoma, (4) did not induce apoptosis of teratoma cells, (5) triggered less mTORC1- /MMP-activation in the tumour microenvironment, (6) and resulted in less attracted murine cells in the teratoma. In summary, these findings provide evidence that human stem cells use the cellular microenvironment to recruit different types of cells to support growth during tumour development.

Since their discovery, human ESCs and iPSCs hold great promise for regenerative medicine. A major risk of pluripotent stem cell-based therapies is the formation of teratomas due to contamination of grafts with remaining undifferentiated cells. The used differentiation protocols are not completely effective and differentiated cell populations contaminated with as few as one hundred ESCs are able to drive teratoma formation^44, 45^. Accordingly, elimination of undifferentiated stem cells is the key issue for the safe clinical application of stem cell-derived transplants. However, none of the currently used elimination strategies, neither cell sorting, nor transfection with suicide genes, antibody-based depletion, small molecule inhibitors, or specific cytotoxic antibodies, has been proven to be able to guarantee stem cell-free transplants^14, 46, 47^. We believe our data to add two new aspects to this ongoing discussion. First, our finding that stem cells can induce invasion of host cells could cause pathological consequences other than teratoma formation. For example, this stem cell property might trigger adverse effects on tissue homoeostasis via dissemination of otherwise stationary cells and remodelling of the ECM^32^. Both phenomena are involved in the development of various types of fibrosis^48, 49^. In line with this, MMP14, which we identified as being a mediator of the stem cell-induced invasive phenotype, has been implicated in the pathogenesis of pulmonary fibrosis^50^. Taken together, we suggest that such consequences of this newly identified stem cell trait must also be taken into account in the context of the development of clinical therapies. Secondly, our results, that stem cell-mediated invasion depends on aberrant induction of drugable targets, such as mTOR, HIF-1α or MMPs, might open a new window of opportunity to minimise the therapy-associated tumour risk.

In conclusion, the observations presented here highlight that a complete picture of the spectrum of stem cell properties cannot be drawn without a thorough understanding of the stem cell-released signals (‘the language of stem cells’) and the consequences for the interaction with their environment.

## Methods

### Antibodies and reagents and cellular treatments

All antibodies used in this study are listed in Supplementary Table 1. Experiments with Marimastat (Tocris) and Rapamycin (Calbiochem) were performed in the absence (DMSO) or presence of the drug at concentrations of 10 μM and 100 nM, respectively. For transwell invasion experiments upon stem cell co-culture the conventional (continuous) Rapamycin treatment was replaced by a 2 h pulse-treatment with subsequent wash-out from the medium to prevent interference of the drug with the cells in the bottom well. For details of the procedure, see Supplementary Fig. 3c–g. For immunoblotting experiments involving growth factor stimulation cells were pre-treated with Rapamycin for 30 min. Human recombinant IGF-I and IGF-II (Peprotech) were used at a concentration of 50 ng ml^−1^. For immunoblotting experiments involving short-term stimulation with defined growth factors or conditioned medium cells were starved in serum-free medium for 12–16 h and then treated and stimulated as indicated. For neutralisation experiments stem cells in the bottom well were washed twice with basal medium and re-fed with complete growth medium containing an IGF-I neutralising antibody or an equal amount of control-IgG. MMP14-dependent activation of pro-MMP2 was assessed via treatment with Concanavalin A (Sigma, 40 μg ml^−1^) for 6 h. To monitor apoptotic cleavage of caspase-3 in cell and tissue lysates, control cell extracts were prepared by treating cells with 1 μM Staurosporine for 3 h.

### Stem cell culture

Human, feeder-independent ESC and iPSC lines with verified normal karyotype were purchased from the Wisconsin International Stem Cell Bank (WiCell Research Institute, Madison, WI, USA) and ATCC and are the following (listed according to Fig. 1a including company, lot/cat. number and passage number upon receipt): ESC-WA01, alias H1 (WiCell, lot no.WA01-DDL-17, p30), ESC-WA09, alias H9 (WiCell, lot no. WB0090, p24), ESC-WA19 (WiCell, lot no. WB0015, p7), iPSC-IMR90-1 (WiCell, lot no. iPS(IMR90)-1-DL-01, p39), iPSC-IMR90-3 (WiCell, lot no.WB0057, p30), iPSC-Foreskin-2 (WiCell, lot no.WB0031, p16), iPSC-Foreskin-4 (WiCell, lot no.WB0038, p19), iPSC-DF6-9-9T.B (WiCell, lot no. Df6-9-9T.B-MCB-01, p23), iPSC-DF19-9-7T (WiCell, lot no. DF19-9-7T-MCB-01, p28), iPSC-DYR0100 (ATCC, cat. no. ACS-1011, lot no. 0190, p24) and iPSC-HYR0103 (ATCC, cat. no. ACS-1007, lot no. 0189, p19). ESC-WA19 were established at the WiCell Research Institute, ESC-WA01 and-WA09 originate from the laboratory of James Thomson (University of Wisconsin, Madison, WI, USA)^5^. iPSC-IMR90-1 and -3 and iPSC-Foreskin-2 and -4 correspond to two independent clones from the same reprogramming experiment, iPSC-DF6-9-9T.B and iPSC-DF19-9-7T are two clones from the same experiment but differ in the combination of episomal expression vectors to achieve reprogramming with OSNLMKT (OCT4, SOX2, NANOG, LIN28, c-MYC, KLF4 and SV40LT)^6, 7^. Purchased ESC and iPSC lines were either derived in defined medium on ECM or transitioned to feeder-independent conditions by the provider. All cells were cultivated according to the WiCell Feeder Independent Pluripotent Stem Cell Protocol (SOP-SH-002, version D) and maintained on Matrigel-coated plates (growth factor-reduced, Corning) in mTeSR1 (Stemcell Technologies) with daily medium change. Cells were grown in colonies at 37 °C and 5% CO_2_ and were routinely passaged with Versene (EDTA, Lonza) every 4 to 6 days at a ratio of 1:10 to 1:20^51^. Colony-type culture was used during regular propagation and throughout experimentation.

Human monoclonal, karyotypically normal AFSC lines AFSC-A1, AFSC-H1 and AFSC-Q1 were generated through immunoselection of c-kit (CD117)-positive cells from human amniotic fluid via magnetic cell sorting, and were provided by Anthony Atala (Wake Forest Institute for Regenerative Medicine, Winston-Salem, NC, USA). AFSCs were maintained on tissue culture-treated plates in Minimal Essential Medium (MEM) α (Invitrogen) supplemented with 15% fetal bovine serum (Hyclone), 18% Chang B, 2% Chang C (both Irvine Scientific) and 2 mM l-Glutamine (Lonza). Cells were grown at 37 °C and 5% CO_2_ and were routinely passaged with Trypsin-EDTA every 2–3 days at a ratio of 1:3 to 1:8.

### Somatic cell and embryonal carcinoma cell culture

Somatic cells and embryonal carcinoma cell lines are the following: CCD1079Sk, primary foreskin fibroblasts (ATCC, CRL-2097, p4); HCF, primary cardiac fibroblasts isolated from the ventricle of an adult heart (Promocell, C-12375, p2); HCH, primary chondrocytes isolated from normal human articular cartilage from the femoral head (Promocell, C-12710, p2); Hep G2, hepatocellular carcinoma cells (ATCC, HB-8065); HFF-1, primary foreskin fibroblasts (ATCC, SCRC-1041, p13); hNHeps, primary hepatocytes isolated from non-transplantable donor tissue (Lonza, CC-2591); HT-1080, fibrosarcoma cells (ATCC, CCL-121, p18); IMR-90, primary lung fibroblasts (ATCC, CCL-186, p10); MCF7, mammary carcinoma cells (ATCC, HTB-22, p146); MEF, immortalised mouse embryonic fibroblasts (provided by David J. Kwiatkowski, Brigham and Women’s Hospital, Harvard Medical School, Boston, MA, USA); NCCIT, embryonal carcinoma cells (ATCC, CRL-2073) and NTERA-2 cl. D1, embryonal carcinoma cells (ATCC, CRL-1973, p18). Experiments in this study were performed with CCD1079Sk between passage 6 and 8, HCF between passage 2 and 4, HCH between passage 2 and 3 and IMR-90 between passage 12 and 16.

CCD1079Sk, Hep G2, HFF-1, HT-1080, IMR-90, MCF7, MEF and NTERA-2 cl. D1 were maintained in Dulbecco’s Modified Eagle Medium (DMEM) at 4.5 g l^−1^ glucose (Invitrogen) supplemented with 10% fetal calf serum (Sigma) and 2 mM l-Glutamine (Lonza), NCCIT in RPMI 1640 (Lonza) containing 10% fetal calf serum and 2 mM l-Glutamine, HCF in Fibroblast Growth Medium 3 (FGM) supplemented with 10% fetal calf serum, basic fibroblast growth factor and insulin (all Promocell), and HCH in Chondrocyte Growth Medium (CGM) supplemented with 10% fetal calf serum (all Promocell). Cells were maintained on tissue culture-treated plates and passaged with Trypsin-EDTA. To maintain a metabolically active state, non-proliferating hNHeps were directly seeded onto Matrigel-coated transwells in Hepatocyte Culture Medium (HCM) (Lonza) containing ascorbic acid, fatty acid free bovine serum albumin, hydrocortisone, epidermal growth factor, transferrin, insulin and gentamicin/amphotericin-B (all Lonza). For initial plating the medium was supplemented with 2% fetal calf serum and changed to serum-free HCM at the first media change after 12 h. All cells were grown at 37 °C and 5% CO_2_.

### Teratoma formation assay

Teratoma formation was performed following standard procedures^52^. In brief, ESC-WA01 (H1) were collected with Versene (EDTA), resuspended in mTeSR1 and mixed with growth factor-reduced Matrigel at a ratio of 1:1. 50 μl of the mixture containing 1 × 10^6^ viable cells were injected intramuscularly into the hind limbs of 8-week-old NOD-SCID IL2Rγ^−/−^ (NSG) mice (The Jackson Laboratory), and tumour growth was monitored by weekly palpation. Control injections were performed with Matrigel alone. Eight weeks after implantation mice were sacrificed and teratomas were dissected. In addition, muscle tissue adjacent to the teratoma (gastrocnemius; ‘Adjacent tissue’ ) and distant muscle tissue with low teratoma proximity (vastus; ‘Neighbouring tissue’) were collected (see also Fig. 3b). All tissue samples were divided into portions, and either formaldehyde-fixed and paraffin-embedded, snap frozen or left untreated for subsequent use in immunofluorescence, immunoblotting and tissue culture. The formation of mature teratomas containing derivatives of all three embryonic germ layers was confirmed by haematoxylin and eosin (H&E) staining of formaldehyde-fixed, paraffin-embedded tissue following standard protocols^53^. To study early events during teratoma formation, mice were killed 4 weeks after injection. Mice were housed under standardised conditions with 12-h photoperiods and ad libitum access to standard diet and water. Animal experiments were approved by the institutional ethics and animal welfare committee and the Austrian government authorities in accordance to the law on animal experimentation. The size of the tumours generated in this study was within the limits allowed in the ethical guidelines of the institution.

### siRNAs, plasmids and transfection

siRNA transfection of primary fibroblasts, AFSCs, ESCs and iPSCs was performed with Lipofectamine RNAiMAX (Invitrogen) as described previously^54^. Pooled siRNAs (mixtures of four different, pre-designed siRNAs targeting one gene) (Dharmacon, ON-TARGETplus SMART pool reagents) were delivered to the cells at a final concentration of 50 nM. A pool of four non-targeting siRNAs was used to control for non-sequence-specific effects. After 48–72 h of incubation, cells were harvested for in vitro analyses. For teratoma assays, siRNA-treated ESCs were collected 24 h after transfection and injected into the hind limbs of NSG mice. The mice were randomly divided into each group without blinding to receive implantation.

Human HIF-1α constructs for the ectopic expression of wildtype and hydroxylation-defective, degradation-resistant HIF-1α were generated in the laboratory of William Kaelin (Dana-Farber Cancer Institute, Boston, MA, USA) and were purchased via Addgene: HA-HIF-1α wildtype in pcDNA3 (Addgene plasmid 18949) and HA-HIF-1α P402A/P564A in pcDNA3 (Addgene plasmid 18955), herein referred to as HIF-1α WT and HIF-1α ΔP, respectively. Plasmids were transfected into primary fibroblasts using Lipofectamine 2000 (Invitrogen). Cells expressing ectopic HIF-1α were identified via co-transfection of GFP-Spectrin at a ratio of 1:5 (GFP-Spectrin:HIF-1α).

### Transwell invasion assay

Invasion of tissue culture cells (in vitro) and collected teratoma/muscle tissues (ex vivo) was assessed via Fluoroblok 24- or 96-well inserts (8.0 μm pore size) pre-coated with standard Matrigel, and pre-coated transparent 24-well inserts (both Corning) for the subsequent detection of invasive cells with Calcein AM (Corning) and crystal violet (Sigma), respectively. For in vitro analyses, the top chambers were seeded with 2.5 × 10^4^ (24-well) or 7.5 × 10^3^ (96-well) target cells resuspended in basal medium (except for hepatocytes which were maintained in complete growth medium). To assess the cell-autonomous invasion of target cells (without stem cell co-culture), bottom chambers were filled with basal medium supplemented with serum or growth factors routinely used for cell maintenance (complete growth medium). For the analysis of stem cell-induced invasion 7.5 × 10^4^–1.0 × 10^5^ (24-well) or 2.25 × 10^4^–3.0 × 10^4^ (96-well) stem cells were seeded into the bottom wells and allowed to grow for 12–24 h. Cells were then washed twice with basal medium and re-fed with complete growth medium before top chambers with target cells were added. Alternatively, 24 h stem cell-conditioned medium was filled in the bottom wells. Wells containing the corresponding stem cell maintenance medium were co-analysed as controls. For the in vitro analysis of invasion and stem cell-induced invasion, target cells in the top chamber were allowed to invade for 22 h. For ex vivo analyses, teratoma/muscle tissue was aseptically harvested, washed twice with DMEM basal medium, minced into equally sized pieces and placed into transwell top chambers. Top chambers and bottom wells were filled with DMEM complete growth medium and invasive outgrowth was monitored after 96 h of incubation.

Invasive cells on the transwell bottom membrane of the Fluoroblok inserts were stained with 4 μg ml^−1^ calcein AM in HBSS (Lonza) for 1 h at 37 °C and 5% CO_2_ and were quantified with a plate reader and/or imaged and counted using an inverted microscope and Photoshop. For transparent inserts, non-invasive cells in the top chamber were removed with cotton swabs, and invasive cells on the transwell bottom membrane were fixed in 4% paraformaldehyde (EMS) and stained with 0.05% crystal violet. Stained cells were scored under the microscope. Independent invasion experiments were performed in duplicate or triplicate wells.

### In vitro plug invasion assay

The plug invasion assay is based on the vertical gel invasion assay^22^ and was performed as previously described^55, 56^ with minor modifications. In brief, growth factor-reduced Matrigel was thawed overnight at 4 °C, and 40–70 μl were directly added to one well of a 96-well plate. Plates were centrifuged at 100 g for 5 min at room temperature followed by an 1 h incubation at 37 °C and 5% CO_2_. Teratoma/muscle tissue was processed as described for the transwell invasion assay, and equally sized pieces were placed on top of the Matrigel plugs, covered with DMEM complete growth medium and incubated for 120 h at 37 °C and 5% CO_2_. For the analysis of the invasive outgrowth, Matrigel plugs were fixed in 2% paraformaldehyde/1% glutaraldehyde (Sigma) for 30–60 min at room temperature and imaged with an inverted microscope. The average length of radial sprouts per sample was quantified using Photoshop and is indicated as the average invasion distance in μm. Invasion was assessed in duplicates.

### Immunofluorescence

Cells in chamber slides were washed with 1× PBS (Lonza) and fixed in 4% paraformaldehyde for 15 min followed by permeabilisation in blocking buffer (1× PBS/1% BSA) containing 0.3% Triton X-100 for 1 h at room temperature and incubation with primary and secondary antibodies diluted in 1× PBS/1% BSA/0.3% Triton X-100 for 12–16 h at 4 °C and 60 min at room temperature, respectively. Nuclei were counterstained with DAPI (2 μg ml^−1^) (Sigma) for 3 min at room temperature. For the staining of paraffin-embedded tissue, 2–3 μm sections were heated to 60 °C for 30 min, followed by deparaffinisation and rehydration in xylene and descending ethanol solutions. Heat-mediated antigen retrieval was performed by boiling in citrate buffer (SignalStain Citrate Unmasking Solution, Cell Signaling). After permeabilisation in 1× PBS/0.1% Triton X-100, sections were blocked in 1× PBS/1% BSA/0.3% Triton X-100 for 1 h at room temperature. Antibody incubation and counterstaining with DAPI was performed as described for the staining of tissue culture cells. Images were taken on a Leica TCS SP8 confocal microscope. For the quantification of Cyclophilin A-positive, murine cells in teratoma tissue, five to eight images per sample were counted using Photoshop. The amount of murine cells was evaluated in relation to 100 teratoma cells.

### Immunoblotting

Cells were washed with 1× PBS, harvested by mild trypsinisation at room temperature and pelleted by centrifugation. Pellets were washed twice with ice-cold 1× PBS and lysed in Triton X-100 buffer (40 mM HEPES, pH 7.5, 120 mM NaCl, 1 mM EDTA, 10 mM β-glycerophosphate, 50 mM NaF, 0.5 mM phenylmethylsulfonyl fluoride, 1% Triton X-100, supplemented with 2 μg ml^−1^ aprotinin, 2 μg ml^−1^ leupeptin, 0.3 μg ml^−1^ benzamidinechloride, 10 μg ml^−1^ trypsin inhibitor) for 20 min on ice. Snap frozen tissue samples were immersed in Triton X-100 buffer and mechanically homogenised (Precellys, Bertin Instruments), followed by two freeze and thaw cycles in liquid nitrogen and 20 min incubation on ice. Supernatants were collected by centrifugation at 20,000 × *g* for 20 min at 4  °C. Equal amounts of denatured samples were resolved by SDS-PAGE and transferred to nitrocellulose following a standard protocol. After blocking with 5% non-fat dry milk for 1 h at room temperature the membrane was incubated with primary antibodies diluted in 5% BSA/1× TBS-T for 12–16 h at 4 °C and secondary antibodies diluted in 5% non-fat dry milk/1× TBS-T for 1 h at room temperature. Signals were detected with the enhanced chemiluminescence method (Pierce). All uncropped western blots can be found in Supplementary Fig. 8.

### Gelatin zymography

Gelatin zymography of tissue culture cell-conditioned medium (in vitro), and tissue-conditioned medium or tissue lysates (ex vivo) was performed following standard protocols^57^. Equal amounts of tissue lysates or 22–24 h conditioned medium were resolved by 10% SDS-PAGE containing 0.2% gelatin (Sigma) in non-reducing sample buffer. Following electrophoresis, SDS was washed out by incubating the gels in renaturation buffer (2.5% Triton X-100) with gentle agitation for 30 min at room temperature. Gels were washed three times in distilled water and were incubated in developing buffer (50 mM Tris-HCl, 200 mM NaCl, 5 mM CaCl_2_, 0.02% Brij 35) for 30 min at room temperature and another 16 h at 37 °C. Gels were stained with 0.5% Coomassie Blue-R-250 for 30 min and destained in 10% methanol/5% acetic acid. For the analysis of tissue-conditioned medium, tissue samples were processed as described for invasion assays and incubated in DMEM basal medium for 24 h at 37 °C and 5% CO_2_. Reverse gelatin zymography for detection of TIMPs was performed as described above except that both, the substrate (0.1% gelatin) and substrate-digesting MMPs (AFSC-conditioned medium), were co-polymerised into the acrylamide gel (15%). Following Coomassie staining, areas of inhibition were visualised as dark bands against a slightly stained background. For experiments in Fig. 7e and Supplementary Fig. 6a, IMR-90- and MCF7-conditioned medium was concentrated using Amicon Ultra centrifugal filter devices (Merck Millipore). All uncropped gel pictures can be found in Supplementary Fig. 8.

### Densitometry

Densitometry for the quantification of signals from immunoblotting and zymography experiments was performed using ImageJ.

### Statistical analysis

Student’s *t*-test (unpaired, two-tailed) was used to compare two groups for independent samples. Values are shown as mean ± standard deviation (s.d.) of biological replicates from independent experiments or one representative experiment. *n* values for each panel in the figures are stated in the corresponding legend. Results documented by immunoblots, zymography gels or micrographs are representative of experiments that were repeated independently.

### Data availability

The authors declare that all data supporting the findings of this study are available within the article and its Supplementary Information files or from the corresponding author upon reasonable request.

## Electronic supplementary material

Supplementary Information
