# Supplementary material for: Human stem cells alter the invasive properties of somatic cells via paracrine activation of mTORC1
Source: Nat Commun. 2017 Sep 19;8:595. doi: 10.1038/s41467-017-00661-x (PMC5605703; doi:10.1038/s41467-017-00661-x)
Supplement: Supplementary file 1 — Supplementary Information [file 41467_2017_661_MOESM1_ESM.pdf]

Description of Supplementary Files

File Name: Supplementary Information
Description: Supplementary Figures, Supplementary Table.

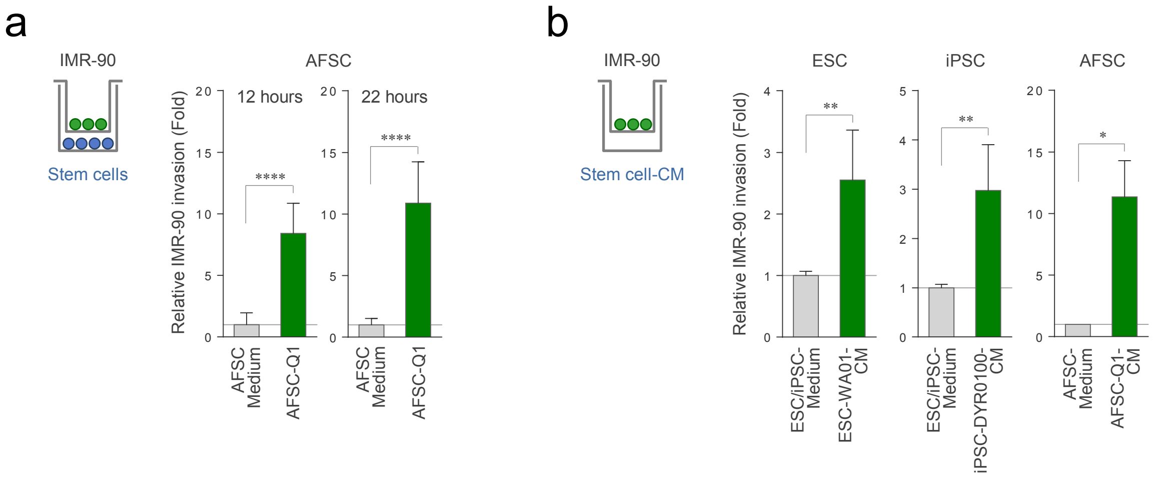

Supplementary Figure 1 Stem cell-induced invasion of primary IMR-90 fibroblasts. Related to Figure 1. (**a**) Transwell invasion assay of IMR-90 fibroblasts upon co-culture with stem cells for 12 and 22 hours ($n \geq 3$; mean ± s.d.). (**b**) Transwell invasion assay of IMR-90 fibroblasts stimulated with stem cell-conditioned medium ($n = 3$; mean ± s.d.). *, $P < 0.05$; **, $P < 0.01$; ****, $P < 0.0001$ by unpaired, two-tailed Student's *t*-test analysis. *n* refers to biological replicates.

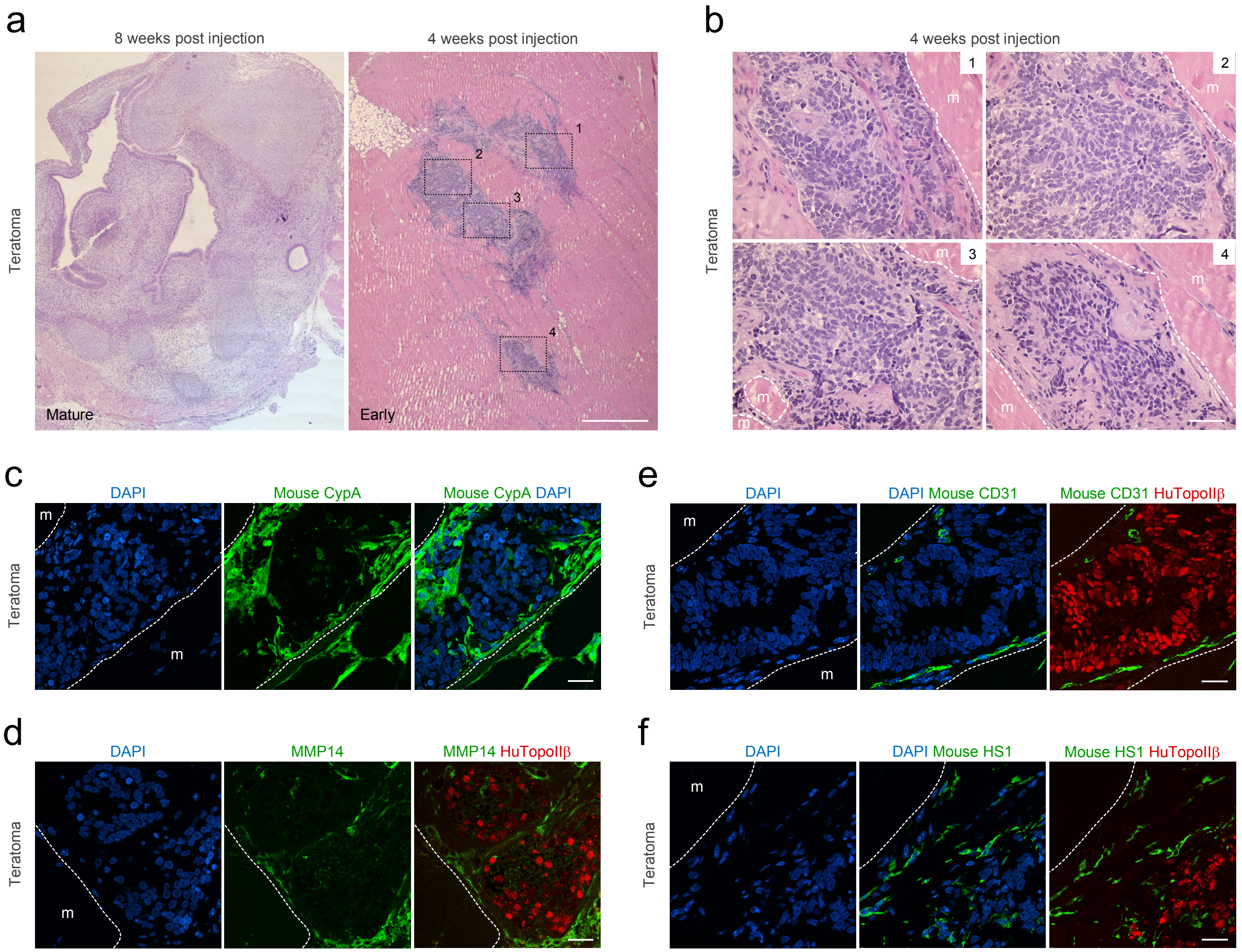

Supplementary Figure 2 ESC-induced invasion during early teratoma formation. Related to Figure 3. (**a**) H&E staining of teratoma tissue at 8 (mature) and 4 (early) weeks after injection. Scale bar, 250 µm. Areas indicated with numbered squares are shown at higher magnification in (**b**), the dotted white line delineates the border between the teratoma and the surrounding muscle tissue (m). Scalebar, 25 µm. Immunostaining of early (4 weeks) teratoma tissue for the detection of mouse cyclophilin A (**c**), MMP14 and human topoisomerase IIβ (**d**), mouse CD31 and human topoisomerase IIβ (**e**), and mouse HS1 and human topoisomerase IIβ (**f**). Nuclei were counterstained with DAPI. The dotted white line delineates the border between the teratoma and the surrounding muscle tissue (m). Scalebars, 25 µm.

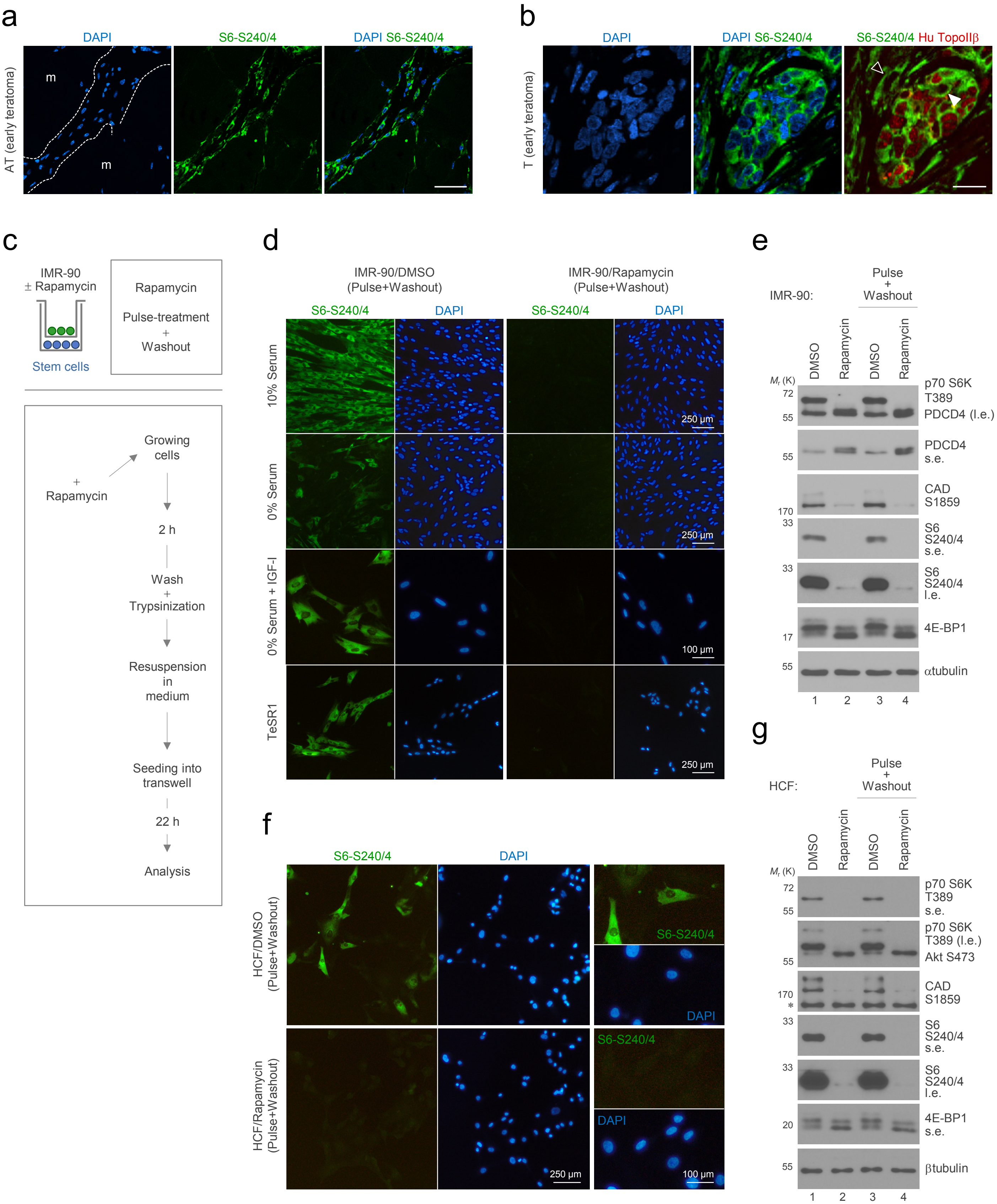

a
DAPI
S6-S240/4
DAPI S6-S240/4
AT (early teratoma)
m
m
b
DAPI
DAPI S6-S240/4
S6-S240/4 Hu TopoIIβ
T (early teratoma)
c
IMR-90
± Rapamycin
Stem cells
Rapamycin
Pulse-treatment
+
Washout
Growing cells
+
Rapamycin
2 h
Wash
+
Trypsinization
Resuspension in medium
Seeding into transwell
22 h
Analysis
d
IMR-90/DMSO (Pulse+Washout)
IMR-90/Rapamycin (Pulse+Washout)
S6-S240/4
DAPI
S6-S240/4
DAPI
10% Serum
0% Serum
0% Serum + IGF-I
TeSR1
250 µm
250 µm
100 µm
250 µm
e
IMR-90:
Pulse + Washout
DMSO
Rapamycin
DMSO
Rapamycin
Mr (K)
72
55
55
170
33
33
17
55
p70 S6K T389
PDCD4 (l.e.)
PDCD4 s.e.
CAD S1859
S6 S240/4 s.e.
S6 S240/4 l.e.
4E-BP1
αtubulin
1
2
3
4
f
S6-S240/4
DAPI
HCF/DMSO (Pulse+Washout)
HCF/Rapamycin (Pulse+Washout)
S6-S240/4
DAPI
S6-S240/4
DAPI
250 µm
100 µm
g
HCF:
Pulse + Washout
DMSO
Rapamycin
DMSO
Rapamycin
Mr (K)
72
55
55
170
*
33
33
20
55
p70 S6K T389 s.e.
p70 S6K T389 (l.e.)
Akt S473
CAD S1859
S6 S240/4 s.e.
S6 S240/4 l.e.
4E-BP1 s.e.
βtubulin
1
2
3
4

Supplementary Figure 3 Stem cell-mediated activation of mTORC1. Related to Figure 4. (**a**) Immunostaining of adjacent tissue from an early (4 weeks) teratoma for the detection of phosphorylated S6. Nuclei were counterstained with DAPI. The dotted white line delineates the border between the invading mouse cells and the surrounding muscle tissue (m). Scalebar, 50 µm. (**b**) Immunostaining of early (4 weeks) teratoma tissue for the detection of phosphorylated S6 and human topoisomerase IIβ. Nuclei were counterstained with DAPI. Arrowheads indicate murine S6 S240/4+ cells between (filled arrowhead) and adjacent to (open arrowhead) human teratoma cells. Scalebar, 20 µm. (**c**) Outline of the Rapamycin pulse-treatment used in transwell co-culture experiments. (**d**) Immunostaining of phosphorylated S6 in IMR-90 fibroblasts pulse-treated with Rapamycin for 22 hours under various growth conditions. Nuclei were counterstained with DAPI. Scale bars, 250 µm and 100 µm. (**e**) Immunoblot for the analysis of mTOR target proteins in Rapamycin-treated IMR-90 fibroblasts. Lanes 1 and 2 correspond to conventional (continuous) treatment, lanes 3 and 4 refer to Rapamycin pulse-treatment described in *c*. (**f**) Immunostaining of phosphorylated S6 in cardiac fibroblasts pulse-treated with Rapamycin for 22 hours. Nuclei were counterstained with DAPI. Scale bars, 250 and 100 µm. (**g**) Immunoblotting for the analysis of mTOR target proteins in Rapamycin-treated cardiac fibroblasts. Lanes 1 and 2 correspond to conventional (continuous) treatment, lanes 3 and 4 refer to Rapamycin pulse-treatment described in *c*. The asterisk next to the CAD S1859 panel indicates a non-specific band.

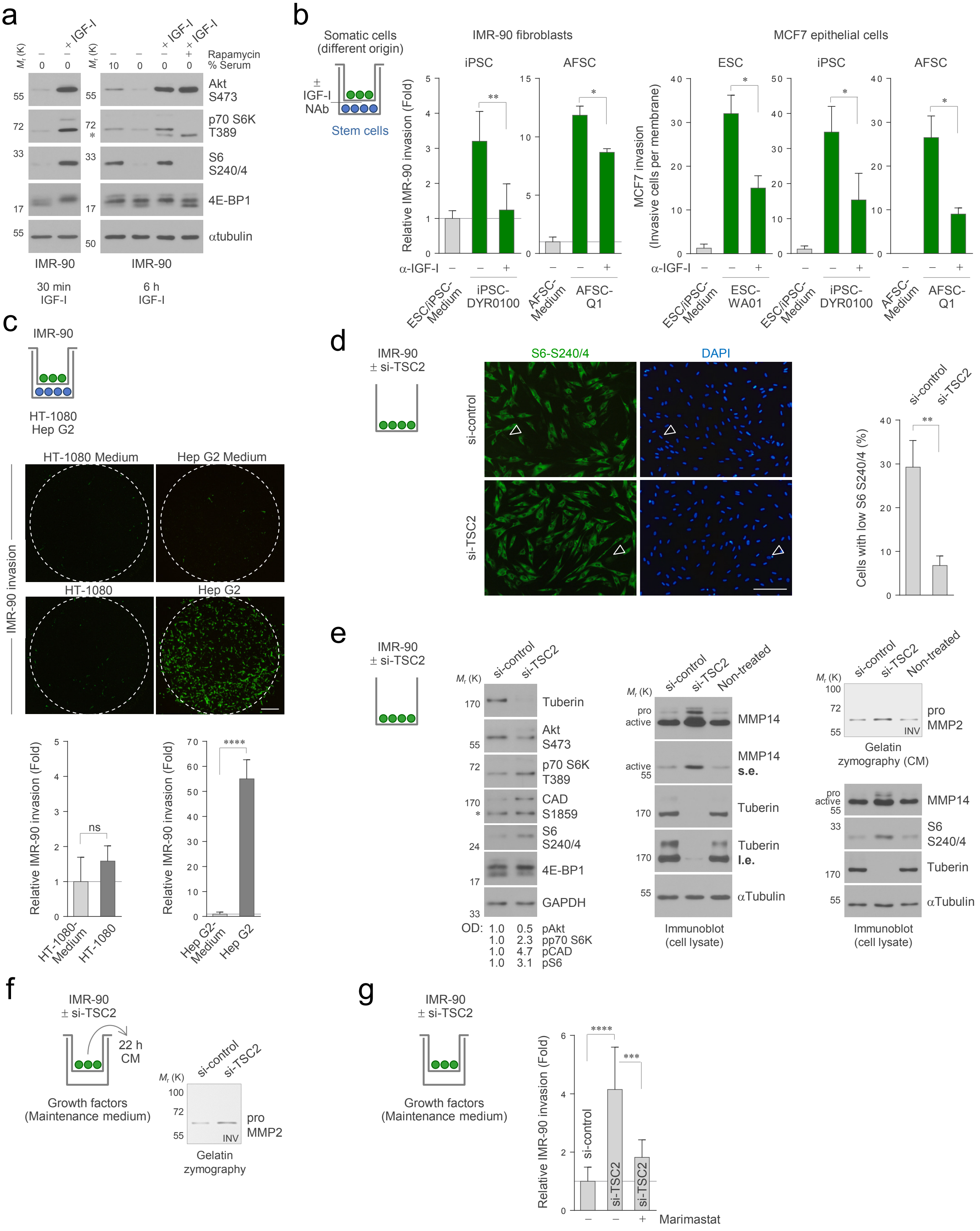

a
IGF-I
Rapamycin
% Serum
Mr (K)
Akt S473
p70 S6K T389
S6 S240/4
4E-BP1
αtubulin
IMR-90
30 min IGF-I
6 h IGF-I
b
Somatic cells (different origin)
± IGF-I NAb
Stem cells
IMR-90 fibroblasts
MCF7 epithelial cells
iPSC
AFSC
ESC
Relative IMR-90 invasion (Fold)
MCF7 invasion (Invasive cells per membrane)
α-IGF-I
ESC/iPSC-Medium
iPSC-DYR0100
AFSC-Medium
AFSC-Q1
ESC-WA01
c
IMR-90
HT-1080
Hep G2
HT-1080 Medium
Hep G2 Medium
IMR-90 invasion
Relative IMR-90 invasion (Fold)
ns
HT-1080-Medium
Hep G2-Medium
d
IMR-90 ± si-TSC2
S6-S240/4
DAPI
si-control
si-TSC2
Cells with low S6 S240/4 (%)
e
Tuberin
CAD S1859
GAPDH
OD: 1.0 0.5 pAkt
1.0 2.3 pp70 S6K
1.0 4.7 pCAD
1.0 3.1 pS6
Non-treated
pro active
MMP14
MMP14 s.e.
Tuberin l.e.
αTubulin
Immunoblot (cell lysate)
pro MMP2
INV
Gelatin zymography (CM)
f
22 h CM
Growth factors (Maintenance medium)
Gelatin zymography
g
Marimastat

Supplementary Figure 4 Activation of the IGF/TSC2/mTOR signalling cascade and its role in cellular invasion. Related to Figure 5. (**a**) Immunoblot analyses of mTOR signalling proteins in serum-deprived IMR-90 cells pre-treated with Rapamycin and stimulated with IGF-I. The asterisk next to the p70 S6K T389 panel indicates a band specific for the prior detection of Akt S473. (**b**) Transwell invasion assay of IMR-90 fibroblasts or MCF7 epithelial cells upon co-culture with stem cells. Stem cell-secreted IGF-I in the bottom well was inhibited via addition of a neutralising antibody ($n \geq 5$, IMR-90; $n \geq 3$, MCF7; mean ± s.d.). NAb, neutralising antibody. (**c**) Transwell invasion assay of IMR-90 fibroblasts upon co-culture with HT-1080 or Hep G2 cells ($n \geq 3$, HT-1080; $n \geq 4$, Hep G2; mean ± s.d.). Pictures show representative Calcein-stainings of invasive cells. Scale bar, 800 µm. (**d**) Immunostaining of phosphorylated S6 in TSC2-depleted IMR-90 fibroblasts. Nuclei were counterstained with DAPI. Arrowheads indicate cells with low expression of phosphorylated S6. A quantification of cells with low S6 activity upon knockdown of TSC2 is included ($n \geq 4$; mean ± s.d.). Scale bar, 500 µm. (**e**) Immunoblotting of cell lysates and gelatin zymography of conditioned medium for the detection of mTOR activity (left panel), MMP14 (middle panel) and secreted MMP2 (right panel) upon knockdown of TSC2. The three panels represent independent experiments. The data in the left panel were densitometrically evaluated (OD). The asterisk next to the CAD S1859 panel indicates a non-specific band. (**f**) Gelatin zymography of transwell top chamber-derived conditioned medium for the analysis of secreted MMP2 in TSC2-depleted IMR-90 cells upon invasion. The gel picture was colour-inverted. (**g**) Transwell invasion assay of TSC2-depleted IMR-90 fibroblasts upon treatment with the broad spectrum MMP-inhibitor Marimastat ($n \geq 8$; mean ± s.d.). *, $P < 0.05$; **, $P < 0.01$; ***, $P < 0.001$; ****, $P < 0.0001$; ns, $P > 0.05$ by unpaired, two-tailed Student's *t*-test analysis. *n* refers to biological replicates.

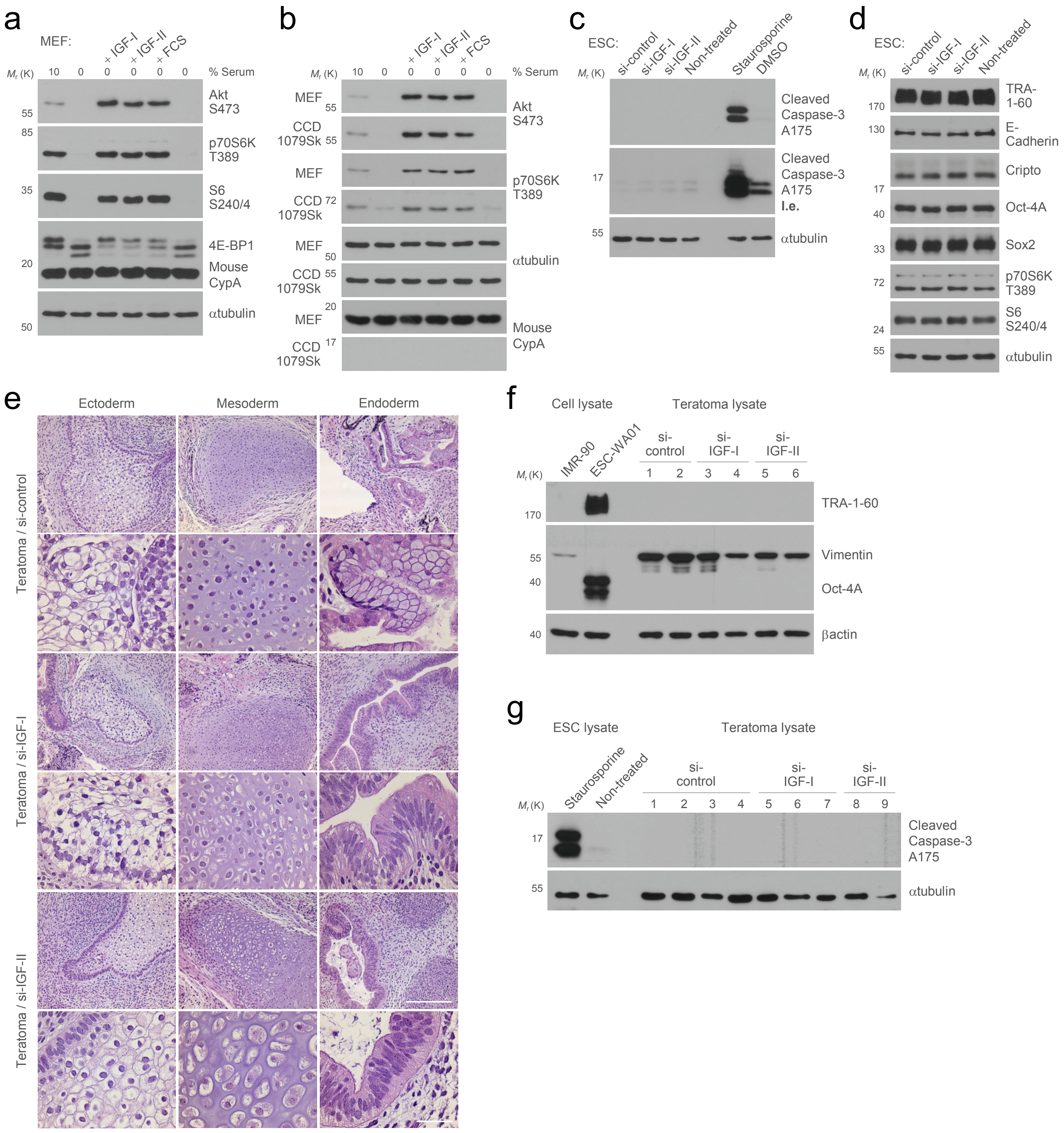

Supplementary Figure 5 Teratoma formation upon knockdown of IGF-I or IGF-II. Related to Figure 6. (**a**) Immunoblot for the detection of mTOR signalling proteins in serum-deprived mouse embryonic fibroblasts stimulated with IGF-I or IGF-II. (**b**) Immunoblot for the detection of mTOR signalling proteins in serum-deprived, IGF-stimulated mouse (MEF) versus human (CCD1079Sk) fibroblasts. (**c**) Immunoblotting for the analysis of cleaved caspase-3 in IGF-I- or IGF-II-depleted ESCs. Staurosporine-treated ESCs were co-analysed as a positive control. (**d**) Immunoblotting for the analysis of pluripotency markers and mTORC1 target proteins in IGF-I- or IGF-II-depleted ESCs. (**e**) H&E staining of teratomas upon injection of IGF-I- or IGF-II-depleted ESCs for the detection of three germ layer-differentiation. Scale bars, 100 and 20 μm. (**f**) Immunoblot of tissue lysates from ESC-si-control- or ESC-si-IGF-injected mice for the detection of pluripotency markers and Vimentin. (**g**) Immunoblot of tissue lysates from ESC-si-control- or ESC-si-IGF-injected mice for the detection of cleaved caspase-3. Lysates of Staurosporine-treated ESCs were co-analysed as a positive control.

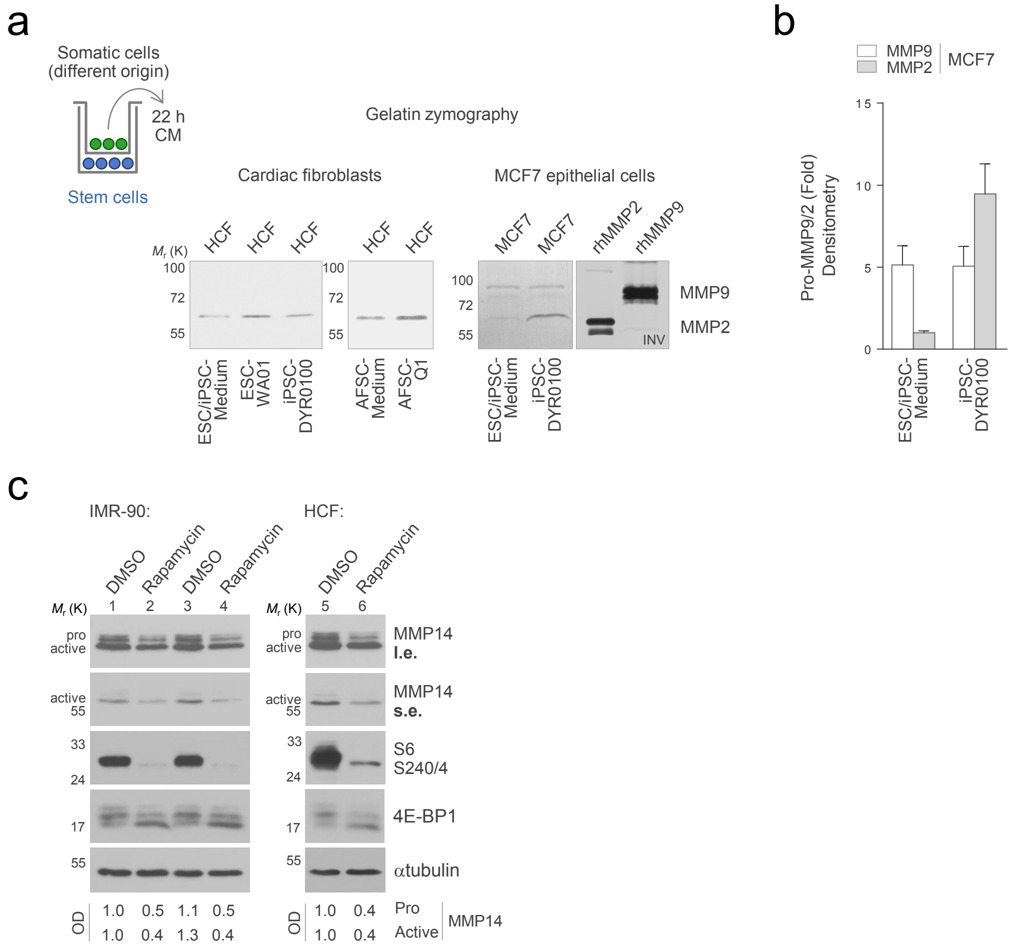

Supplementary Figure 6 Stem cell- and mTORC1-mediated activation of MMPs. Related to Figure 7. (**a**) Gelatin zymography of transwell top chamber-derived conditioned medium for the analysis of secreted MMP2 and MMP9 in cardiac fibroblasts and MCF7 cells co-cultured with stem cells. The gel pictures were colour-inverted. MCF7 results of independent experiments were densitometrically analysed and are shown in (**b**), (n = 2; mean ± s.d.). (**c**) Immunoblots for the analysis of MMP14 in IMR-90 cells and cardiac fibroblasts upon Rapamycin treatment (lanes 1,2, continuous treatment; lanes 3,4,5,6, pulse-treatment). Data were densitometrically evaluated (OD). n refers to biological replicates.

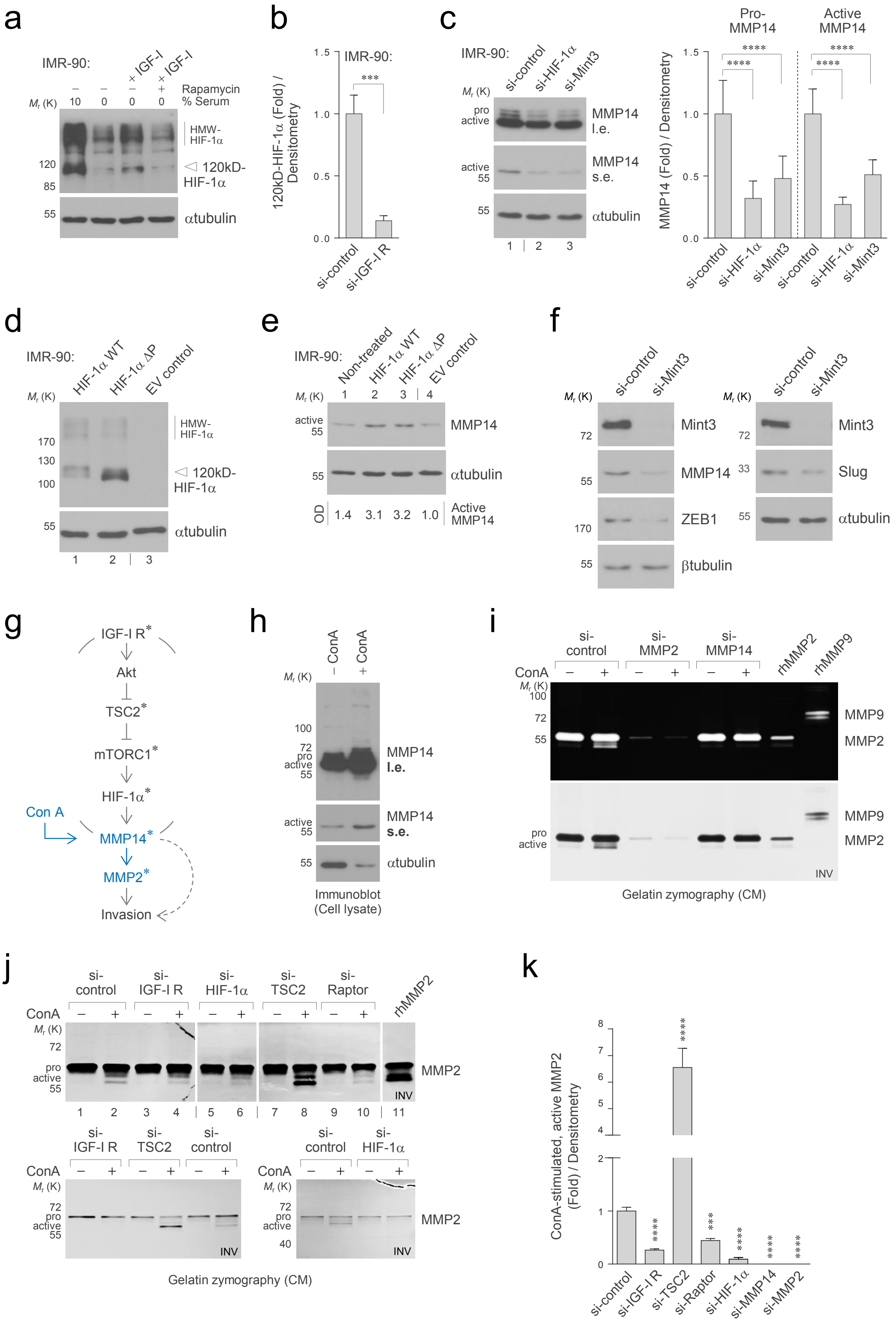
a
IMR-90:
+ IGF-I
+ IGF-I
Rapamycin
% Serum
HMW-HIF-1α
120kD-HIF-1α
αtubulin
b
IMR-90:
120kD-HIF-1α (Fold) / Densitometry
si-control
si-IGF-I R
c
IMR-90:
si-control
si-HIF-1α
si-Mint3
MMP14 l.e.
MMP14 s.e.
αtubulin
Pro-MMP14
Active MMP14
MMP14 (Fold) / Densitometry
d
IMR-90:
HIF-1α WT
HIF-1α ΔP
EV control
HMW-HIF-1α
120kD-HIF-1α
αtubulin
e
IMR-90:
Non-treated
HIF-1α WT
HIF-1α ΔP
EV control
MMP14
αtubulin
OD 1.4 3.1 3.2 1.0 Active MMP14
f
si-control
si-Mint3
Mint3
MMP14
ZEB1
βtubulin
Slug
αtubulin
g
IGF-I R*
Akt
TSC2*
mTORC1*
HIF-1α*
Con A
MMP14*
MMP2*
Invasion
h
– ConA
+ ConA
MMP14 l.e.
MMP14 s.e.
αtubulin
Immunoblot (Cell lysate)
i
si-control
si-MMP2
si-MMP14
rhMMP2
rhMMP9
ConA
MMP9
MMP2
INV
Gelatin zymography (CM)
j
si-control
si-IGF-I R
si-HIF-1α
si-TSC2
si-Raptor
rhMMP2
ConA
MMP2
INV
Gelatin zymography (CM)
k
ConA-stimulated, active MMP2 (Fold) / Densitometry
si-control
si-IGF-I R
si-TSC2
si-Raptor
si-HIF-1α
si-MMP14
si-MMP2

Supplementary Figure 7 Role of the IGF/mTOR signalling cascade for the activation of HIF-1α and MMPs. Related to Figure 8. (**a**) Immunoblot for HIF-1α in serum-deprived IMR-90 cells pre-treated with Rapamycin and stimulated with IGF-I. (**b**) Quantification of HIF-1α in IGF-I receptor-depleted IMR-90 cells via densitometry of immunoblots (n = 3; mean ± s.d.). (**c**) Left panel: Immunoblot for MMP14 upon HIF-1α or Mint3 knockdown in IMR-90 cells. The bar indicates vertical cropping. Right panel: Quantification of MMP14 in HIF-1α- or Mint3-depleted IMR-90 cells via densitometry of immunoblots ($n \geq 6$; mean ± s.d.). (**d**) Immunoblot for the transient overexpression of wildtype and degradation-resistant HIF-1α in IMR-90 fibroblasts. The bar indicates vertical cropping. (**e**) MMP14 expression upon HIF-1α overexpression via immunoblotting of IMR-90 lysates. Data were densitometrically evaluated (OD). The bar indicates vertical cropping. (**f**) Immunoblots for the expression of HIF-1α target proteins in Mint3-depleted IMR-90 fibroblasts. (**g**) The IGF-I/Akt/mTORC1 signalling cascade and its suggested involvement in the regulation of MMP2. Asterisks indicate experimentally modified proteins. (**h**) Immunoblotting of cell lysates for the analysis of MMP14 in IMR-90 fibroblasts treated with Concanavalin A. (**i**) Gelatin zymography of conditioned medium for the analysis of secreted MMP2 in IMR-90 cells treated with Concanavalin A. (**j**) Gelatin zymography of conditioned medium for the analysis of secreted MMP2 in IMR-90 cells depleted of mTOR signalling proteins and treated with Concanavalin A. The three panels (upper, lower left and lower right) correspond to three independent experiments. The bar indicates vertical cropping. (**k**) Densitometry of zymography gels for the quantification of active MMP2 in IMR-90 cells upon siRNA- and Concanavalin A treatment ($n \geq 3$; mean ± s.d.). ***, $P < 0.001$; ****, $P < 0.0001$ by unpaired, two-tailed Student's t-test analysis. n refers to biological replicates.

Fig. 3e

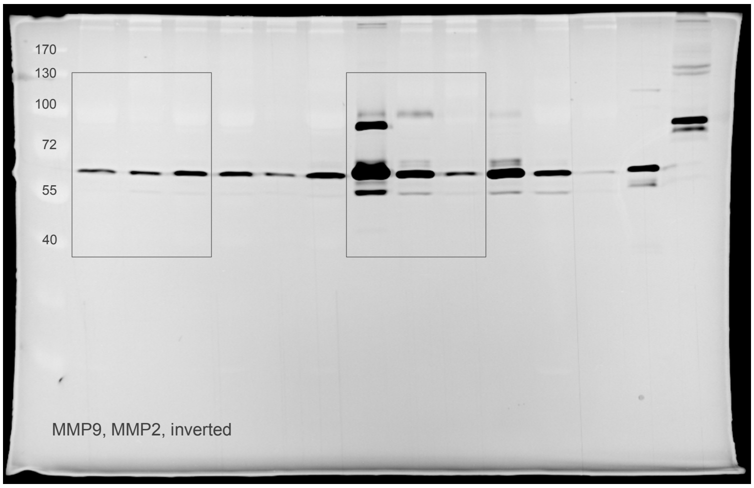

Fig. 3f

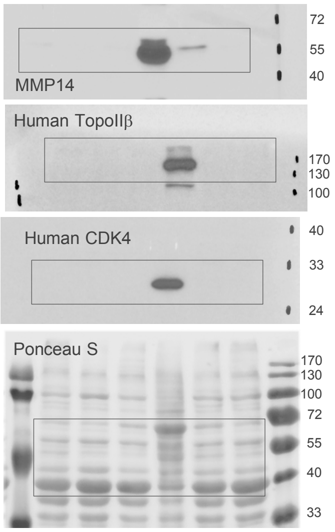

Fig. 3k

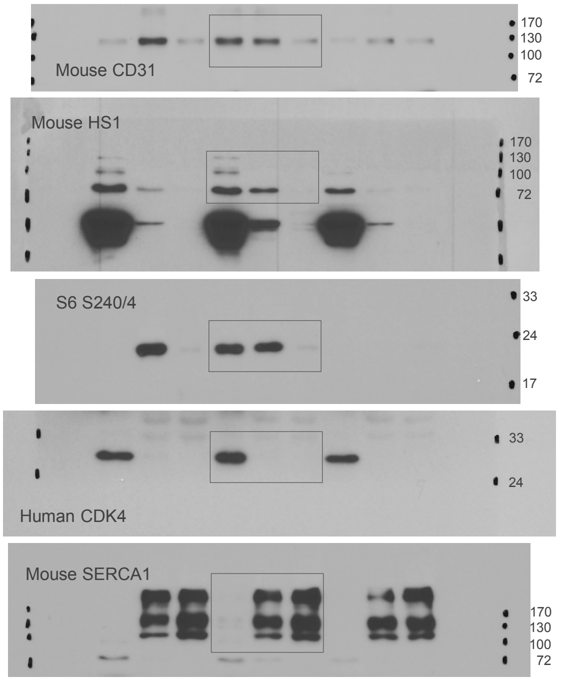

Fig. 4d

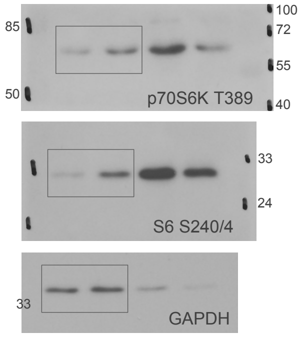

Fig. 4e

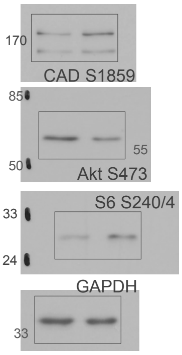

Fig. 4f

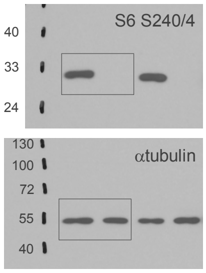

Fig. 4h

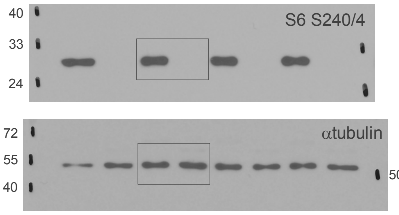

Fig. 5a

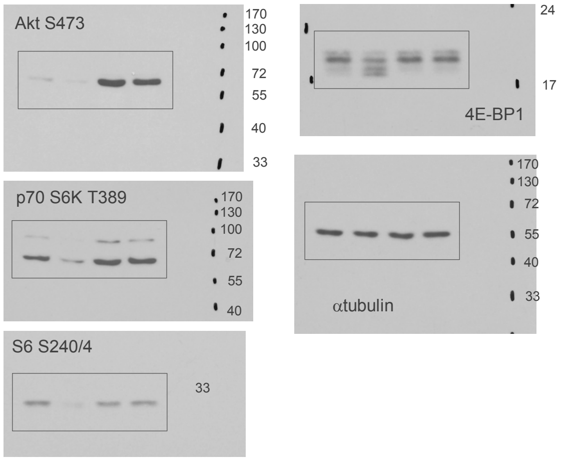

Fig. 5c

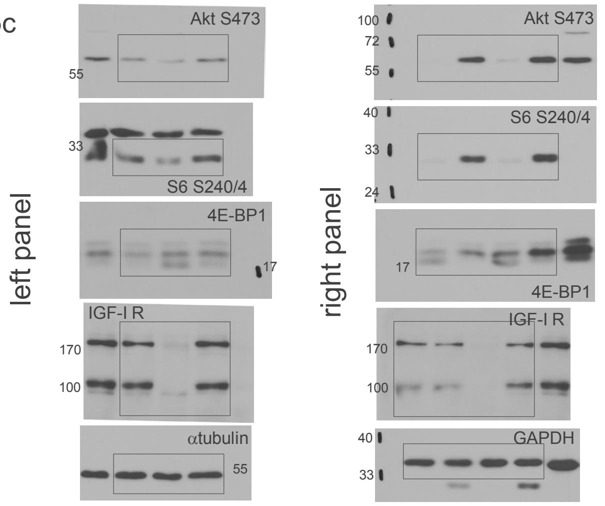

Fig. 5e

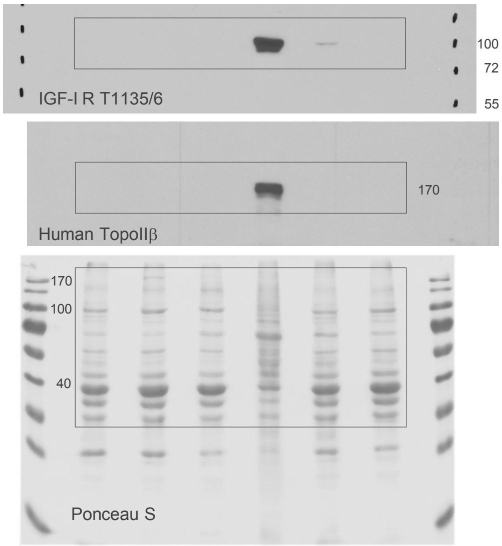

Fig. 6d

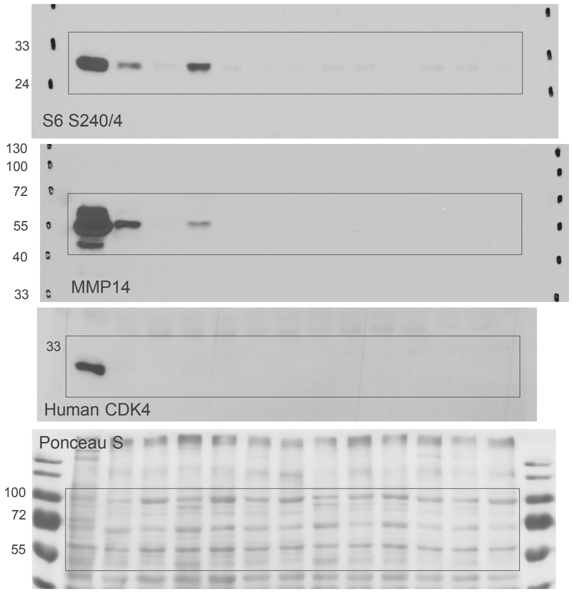

Fig. 6e

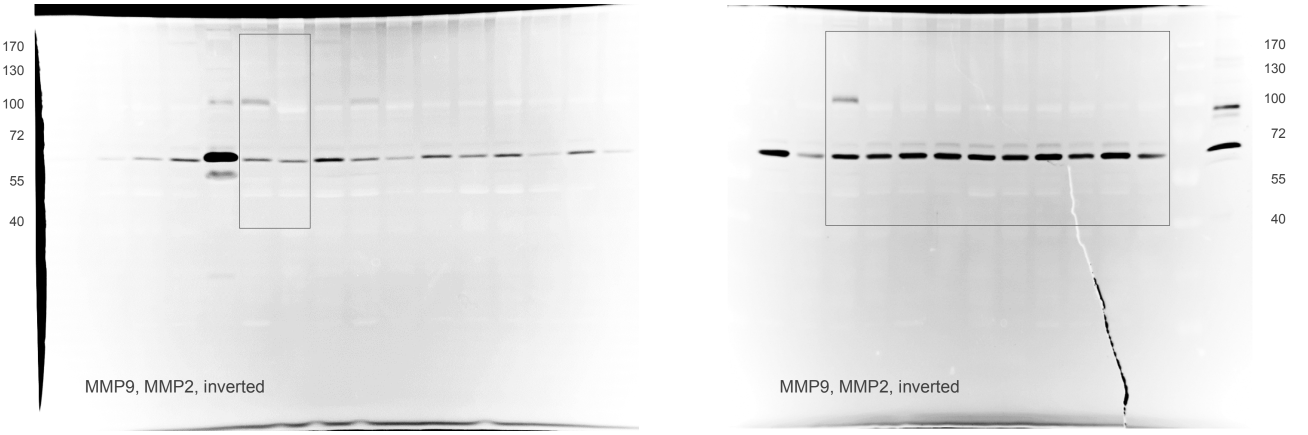

Fig. 7a

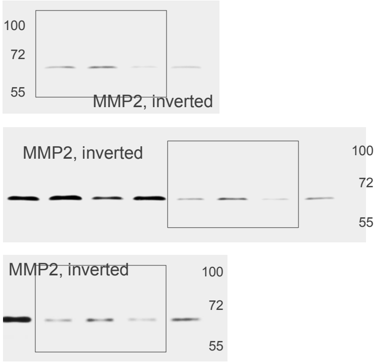

Fig. 7c

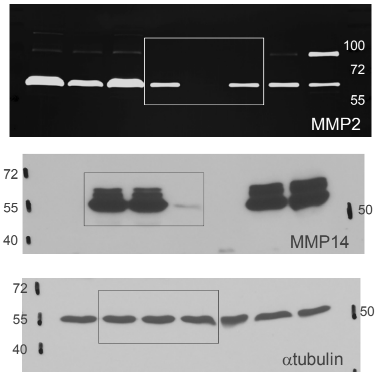

Fig. 7d

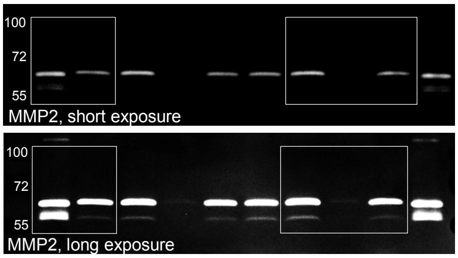

Fig. 7e

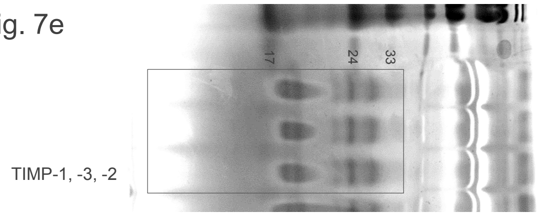

Fig. 8a

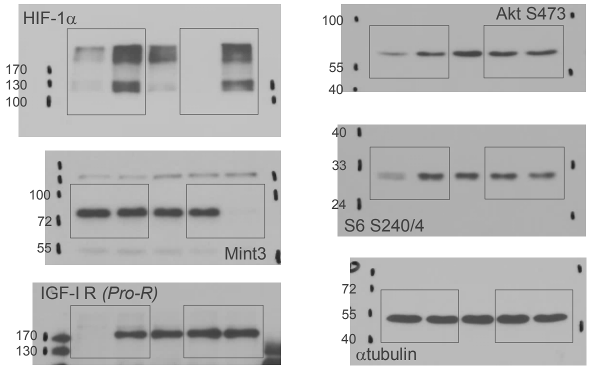

Fig. 8b

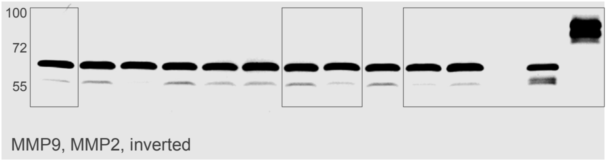

Fig. 8d

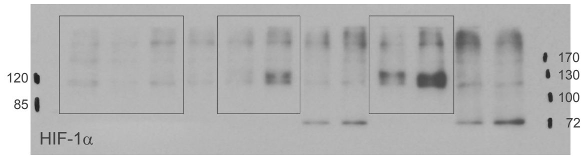

Fig. 8d continued

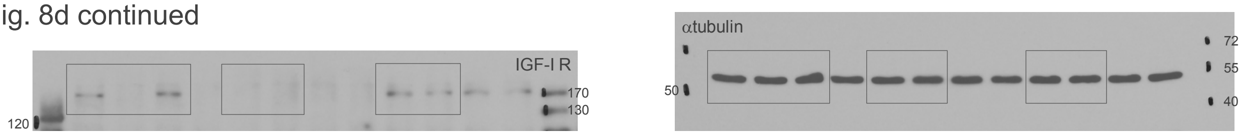

Suppl.Fig. 3e

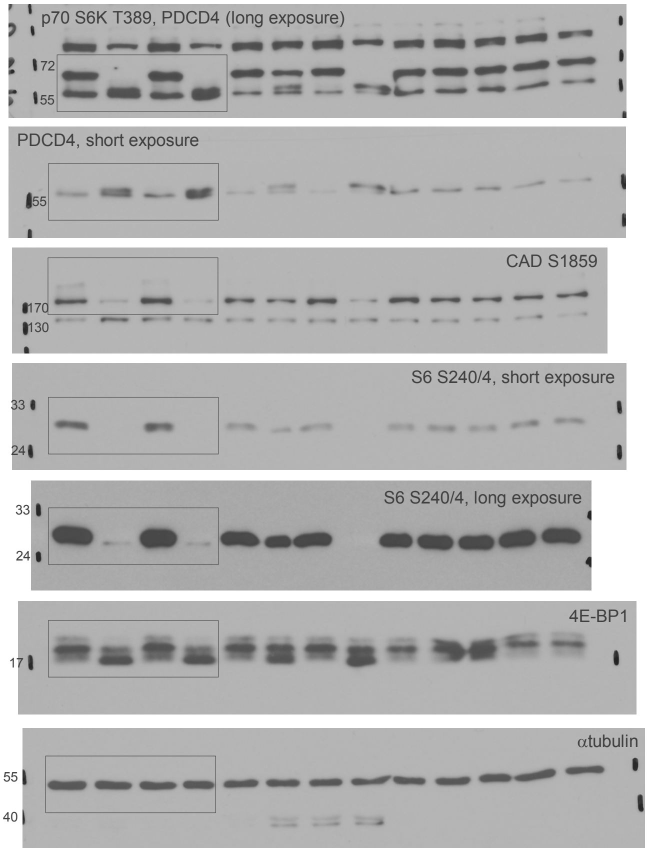

Suppl.Fig. 3g

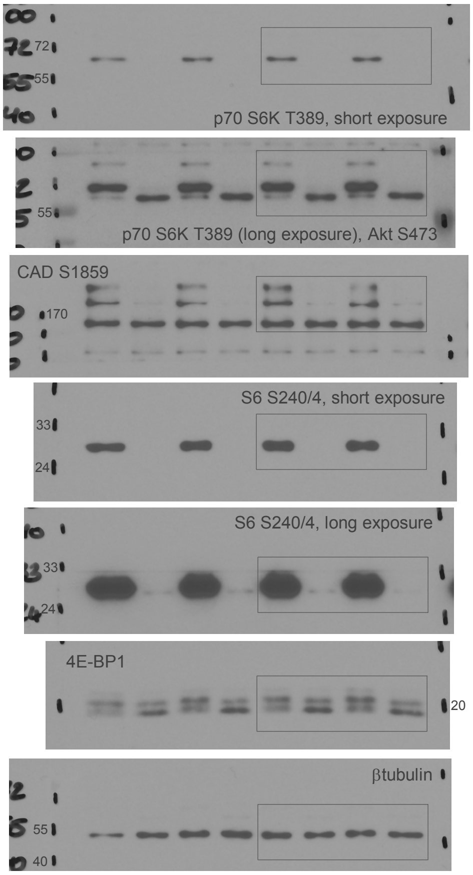

Suppl.Fig. 4a

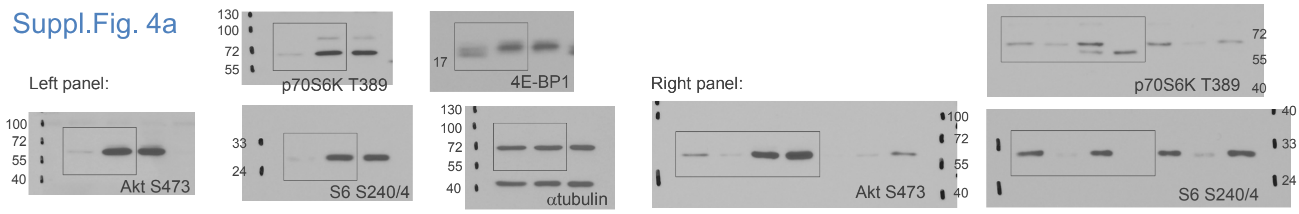

Suppl.Fig. 4a continued

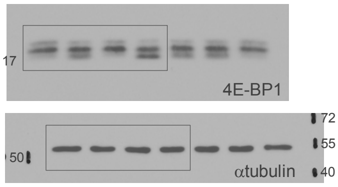

Suppl.Fig. 4e

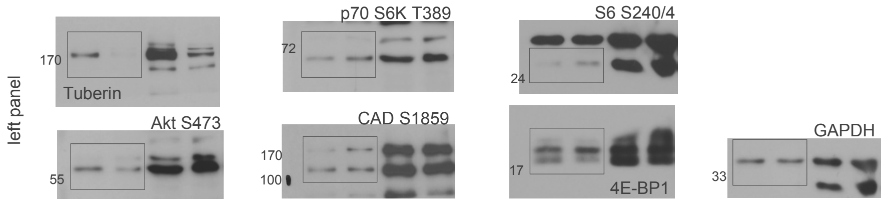

Suppl.Fig. 4e continued

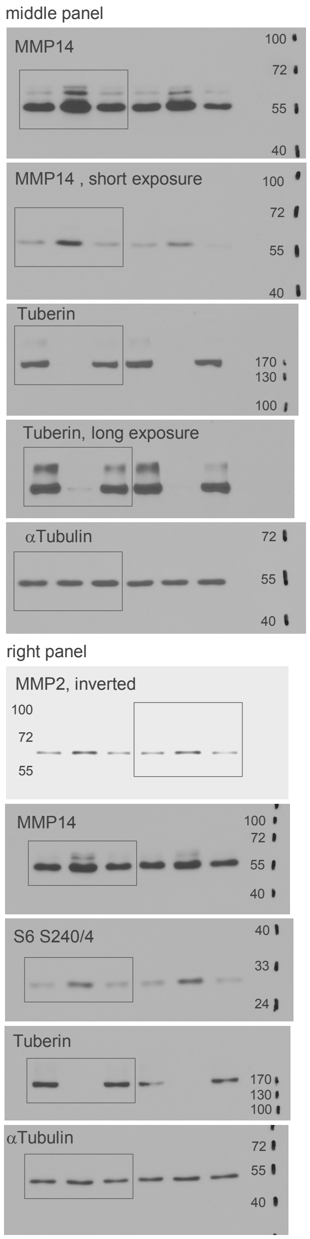

Suppl.Fig. 4f

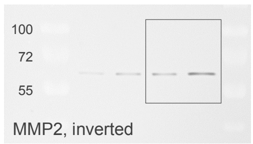

Suppl.Fig. 5a

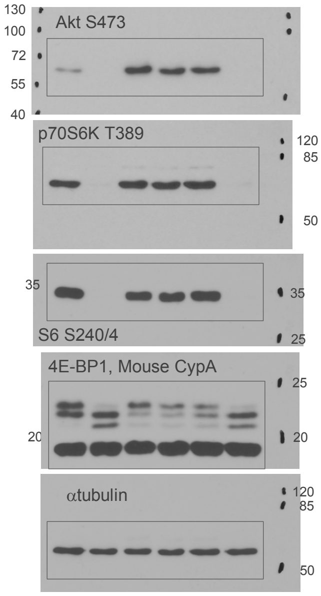

Suppl.Fig. 5b

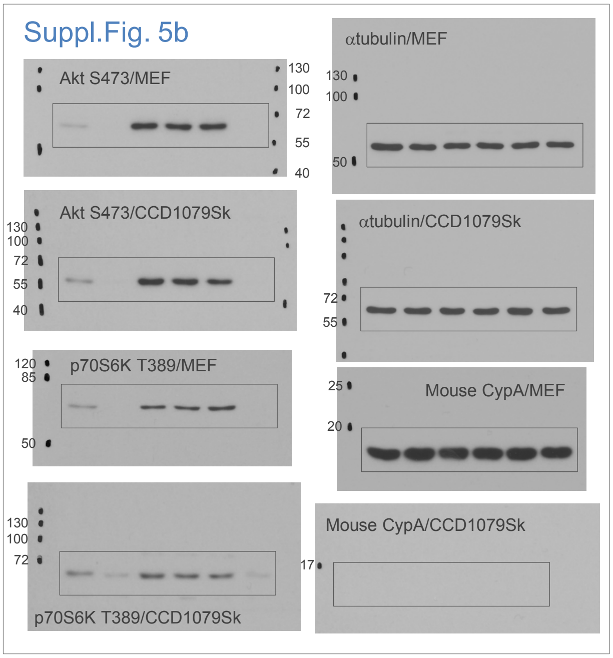

Suppl Fig. 5c

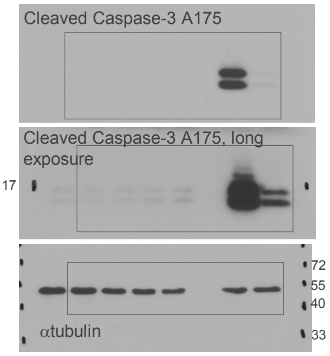

Suppl.Fig. 5d continued

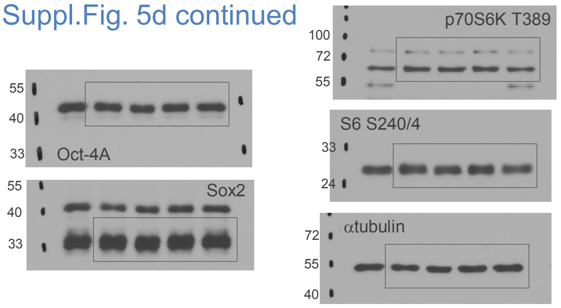

Suppl.Fig. 5d

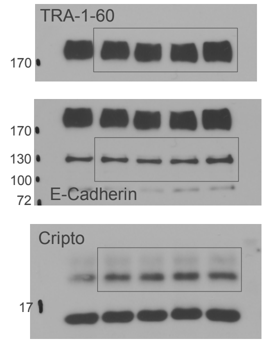

Suppl.Fig. 5f

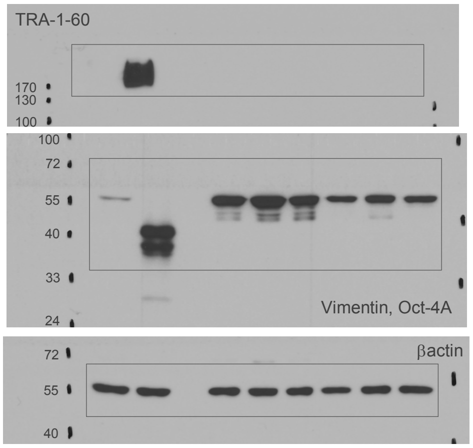

Suppl.Fig. 5g

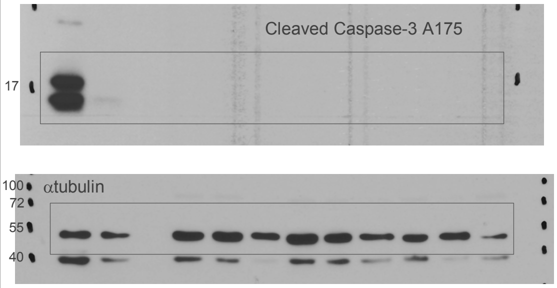

Suppl.Fig. 6c

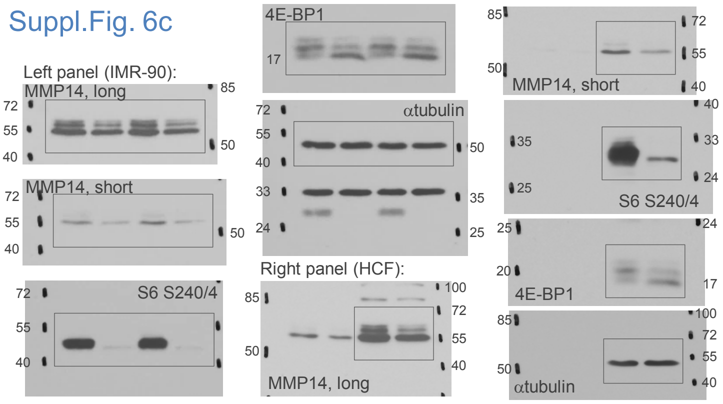

Suppl.Fig. 6a

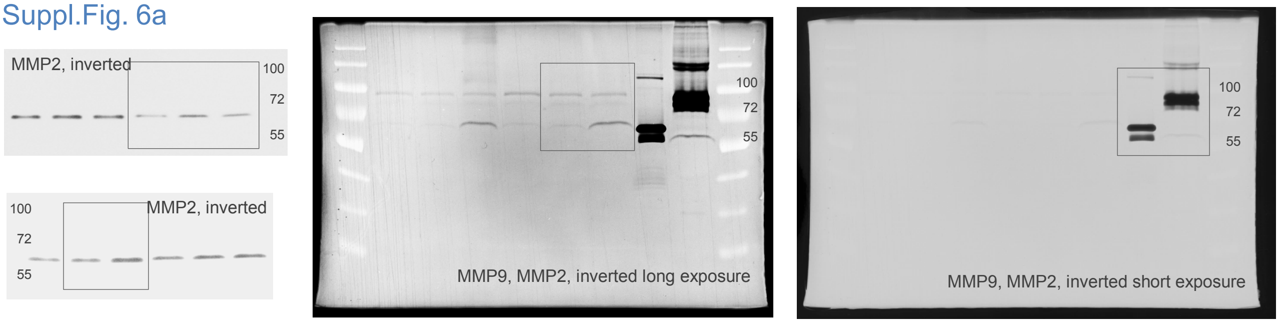

Suppl.Fig. 7a

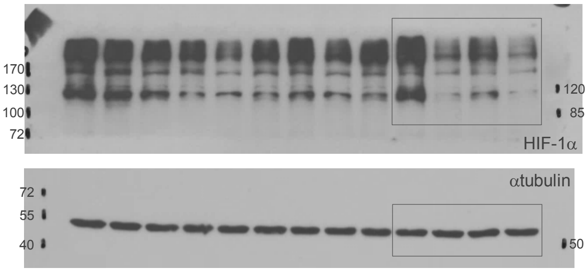

Suppl.Fig. 7c

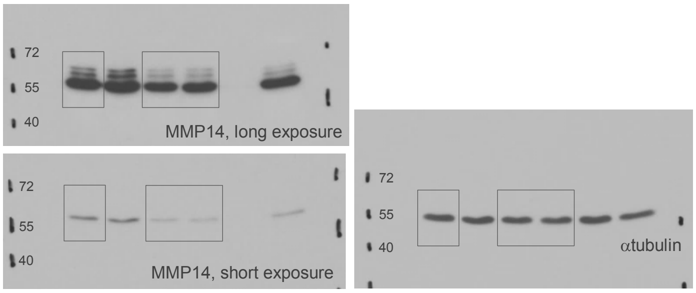

Suppl.Fig. 7d

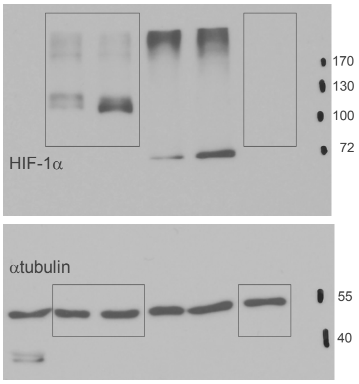

Suppl.Fig. 7e

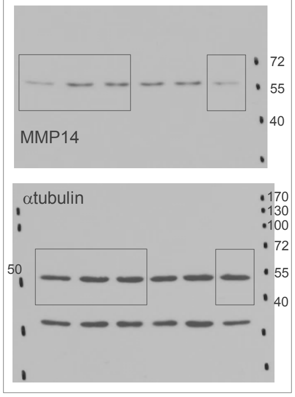

Suppl.Fig. 7f

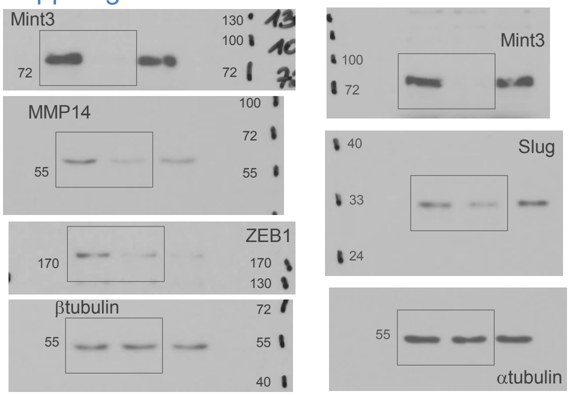

Suppl.Fig. 7h

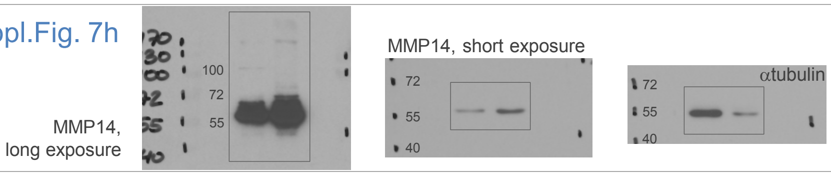

Suppl.Fig. 7i

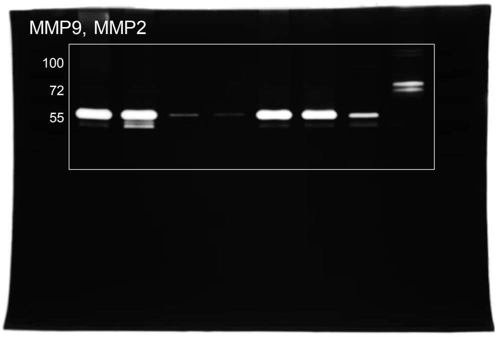

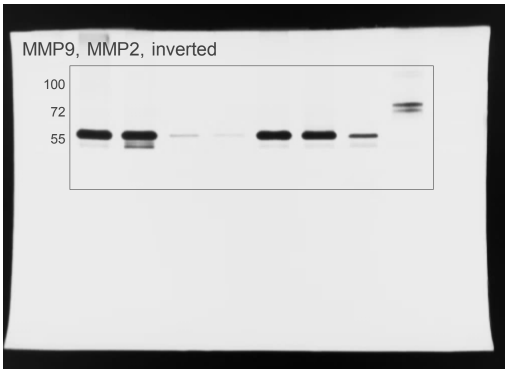

Suppl.Fig. 7j

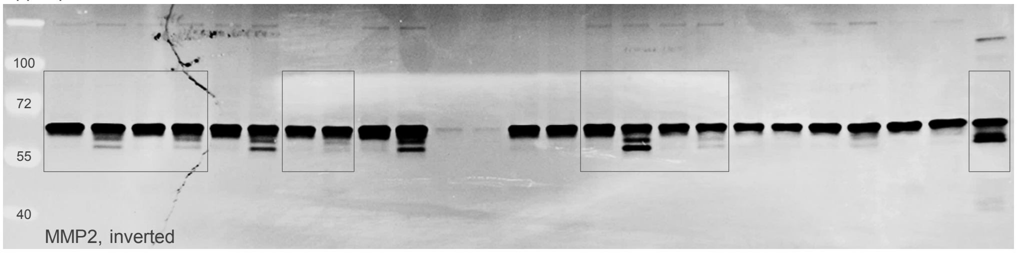

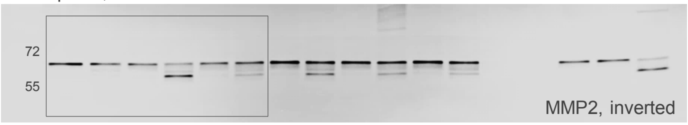

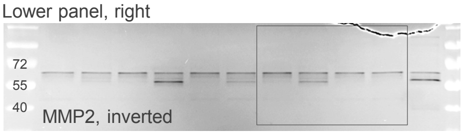

Supplementary Figure 8 Uncropped scans of immunoblots and zymography gels. Related to main and supplementary figures. The corresponding figure number and the molecular weight in kDa are indicated. Gray boxes highlight the cropped areas presented in the figures.

Supplementary Table 1 Primary and secondary antibodies used in this study.

Antigen	Clone No.	Company	Cat No.	Dilution/Concentration			
				IB	IF (TC)	IF (P)	N
4E-BP1	53H11	Cell Signaling	9644	1:2000			
Akt S473	D9E	Cell Signaling	4060	1:1000			
CAD S1859		Cell Signaling	12662	1:1000			
CD31 (PECAM-1)	D8V9E	Cell Signaling	77699	1:1000		1:100	
CDK4	D9G3E	Cell Signaling	12790	1:500			
Cleaved caspase-3 A175		Cell Signaling	9661	1:1000			
Cripto	D81B12	Cell Signaling	4193	1:1000			
Cyclophilin A	D2Y4M	Cell Signaling	51418	1:1000		1:100	
E-cadherin	36	BD Transduction Laboratories	610181	1:1000			
GAPDH	D16H11	Cell Signaling	5174	1:1000			
GAPDH		Trevigen	2275	1:10000			
HIF-1α	54	BD Transduction Laboratories	610958	1:250			
HS1	D5A9	Cell Signaling	3892	1:1000		1:100	
IGF-I		Abcam	ab9572		1:200		
IGF-I		R&D Systems	AF-291-NA				25 ng ml^{-1}
IGF-I R β T1135/1136 / Insulin R β T1150/1151	19H7	Cell Signaling	3024	1:500			
IGF-I R β	D23H3	Cell Signaling	9750	1:1000			
IGF-II	S1F2	Merck Millipore	05-166		1:200		
Mint3	32	BD Transduction Laboratories	611380	1:1000			
MMP14	EP1264Y	Abcam	ab51074	1:5000		1:100	
Oct-4A	C30A3	Cell Signaling	2840	1:2000			
p70 S6K T389	108D2	Cell Signaling	9234	1:1000			
PDCD4	D29C6	Cell Signaling	9535	1:1000			
S6 S240	DAK-S6-240	Agilent Technologies/DAKO	M730029-8			1:50	
S6 S240/4	D68F8	Cell Signaling	5364	1:5000	1:800		
SERCA1	A988	Cell Signaling	4274	1:2000			
Slug	C19G7	Cell Signaling	9585	1:1000			
SMA	1A4	Abcam	ab7817			1:100	
Sox2	D6D9	Cell Signaling	3579	1:1000			
Topoisomerase IIβ	40	BD Transduction Laboratories	611493	1:500		1:50	
TRA-1-60(S)	TRA-1-60(S)	Cell Signaling	4746	1:1000			
Tuberin/TSC2	D93F12	Cell Signaling	4308	1:1000			
Vimentin	D21H3	Cell Signaling	5741	1:1000			
ZEB1/TCF8	D80D3	Cell Signaling	3396	1:1000			
αtubulin	DM1A	Calbiochem	CP06	1:5000			
βactin	C4	Santa Cruz	47778	1:1000			
βtubulin	9F3	Cell Signaling	2128	1:1000			
rabbit IgG (H+L), HRP		Cell Signaling	7074	1:5000			
mouse IgG (H+L), HRP		Cell Signaling	7076	1:5000			
rabbit IgG (H+L), Alexa 488		Cell Signaling	4412		1:500	1:500	
mouse IgG (H+L), Alexa 594		Cell Signaling	8890		1:500	1:500	

Abbreviations: IB, immunoblotting; IF (TC), immunofluorescence staining of tissue culture cells; IF (P), immunofluorescence staining of paraffin-embedded tissue; N, neutralisation.
